# Antigenic evolution of human influenza H3N2 neuraminidase is constrained by charge balancing

**DOI:** 10.7554/eLife.72516

**Published:** 2021-12-08

**Authors:** Yiquan Wang, Ruipeng Lei, Armita Nourmohammad, Nicholas C Wu

**Affiliations:** 1 Department of Biochemistry, University of Illinois at Urbana-Champaign Urbana United States; 2 Department of Physics, University of Washington Seattle United States; 3 Max Planck Institute for Dynamics and Self-Organization Göttingen Germany; 4 Fred Hutchinson Cancer Research Center Seattle United States; 5 Center for Biophysics and Quantitative Biology, University of Illinois at Urbana-Champaign Urbana United States; 6 Carl R. Woese Institute for Genomic Biology, University of Illinois at Urbana-Champaign Urbana United States; 7 Carle Illinois College of Medicine, University of Illinois at Urbana-Champaign Urbana United States; University of Basel Switzerland; The Feinstein Institute for Medical Research United States

**Keywords:** influenza, neuraminidase, evolution, epistasis, combinatorial mutagenesis, next-generation sequencing, Influenza virus

## Abstract

As one of the main influenza antigens, neuraminidase (NA) in H3N2 virus has evolved extensively for more than 50 years due to continuous immune pressure. While NA has recently emerged as an effective vaccine target, biophysical constraints on the antigenic evolution of NA remain largely elusive. Here, we apply combinatorial mutagenesis and next-generation sequencing to characterize the local fitness landscape in an antigenic region of NA in six different human H3N2 strains that were isolated around 10 years apart. The local fitness landscape correlates well among strains and the pairwise epistasis is highly conserved. Our analysis further demonstrates that local net charge governs the pairwise epistasis in this antigenic region. In addition, we show that residue coevolution in this antigenic region is correlated with the pairwise epistasis between charge states. Overall, this study demonstrates the importance of quantifying epistasis and the underlying biophysical constraint for building a model of influenza evolution.

## Introduction

There are two major antigens on the surface of influenza virus, hemagglutinin (HA) and neuraminidase (NA). Although influenza vaccine development has traditionally focused on HA, NA has emerged as an effective vaccine target in the past few years because recent studies have shown that NA immunity has a significant role in protection against influenza infection (Monto et al., 2015; Weiss et al., 2020; Couch et al., 2013; Memoli et al., 2016; Krammer et al., 2018). Influenza NA has an N-terminal transmembrane domain, a stalk domain, and a C-terminal head domain. The head domain of NA functions as an enzyme to cleave the host receptor (i.e., sialylated glycan), which is essential for virus release. Most NA antibodies target the surface loop regions that surround the highly conserved catalytic active site (Gulati et al., 2002; Malby et al., 1994; Venkatramani et al., 2006; Colman et al., 1983; Air, 2012; Zhu et al., 2019; Tulip et al., 1992). Due to the need to constantly escape from herd immunity (also known as antigenic drift), both HA and NA of human influenza virus have evolved extensively (Kilbourne et al., 1990; Sandbulte et al., 2011; Westgeest et al., 2015). For example, since influenza H3N2 virus entered the human population in 1968, its HA and NA have accumulated more than 83 and 73 amino acid mutations, respectively (Figure 1—figure supplement 1), which accounted for ~15% of their protein sequences. However, the evolution of NA is much less well characterized as compared to HA.

To understand how the evolutionary trajectories of NA are being shaped, it is important to characterize the underlying biophysical constraints that govern the fitness of individual amino acid mutations and epistatic interactions between mutations (Starr and Thornton, 2016; Echave and Wilke, 2017; Gong et al., 2013). Epistasis is a phenomenon in which the fitness effect of a mutation is dependent on the presence or absence of other mutations. Since epistasis can lead to differential fitness effects of a given mutation on different genetic backgrounds, it can restrict evolutionary trajectories or open up a new functional sequence space that would otherwise be inaccessible (Wu et al., 2016). As a result, epistasis is a primary challenge for predicting evolution (Miton and Tokuriki, 2016; Luksza and Lässig, 2014). Nevertheless, epistasis is pervasive in natural evolution in general (Breen et al., 2012) and has been shown to influence the evolution of influenza virus (Gong et al., 2013; Wu et al., 2016; Lyons and Lauring, 2018; Wu et al., 2018; Kryazhimskiy et al., 2011; Wu et al., 2020; Koel et al., 2019; Bloom et al., 2010; Abed et al., 2011; Wu et al., 2013; Duan et al., 2014). While epistasis is critical for the emergence of oseltamivir-resistant mutants of influenza NA (Bloom et al., 2010; Abed et al., 2011; Wu et al., 2013; Duan et al., 2014), the role of epistasis and the underlying biophysical constraints on NA antigenic evolution remains largely unclear.

Deep mutational scanning combines saturation mutagenesis and next-generation sequencing to determine the phenotypic effects of numerous mutations in a highly parallel manner (Lee et al., 2018; Narayanan and Procko, 2021; Fowler and Fields, 2014). Deep mutational scanning has been employed to measure the replication fitness effect of all possible single amino acid mutations across different influenza proteins (Lee et al., 2018; Thyagarajan and Bloom, 2014; Doud et al., 2015; Soh et al., 2019; Hom et al., 2019), which in turn can help to model the natural evolution of human influenza virus (Lee et al., 2018; Thyagarajan and Bloom, 2014). However, most deep mutational scanning experiments lack the power to systematically measure the fitness of variants with two or more mutations, hence epistasis. More recently, combinatorial mutagenesis was used in conjunction with next-generation sequencing to examine the local fitness landscape and to identify epistasis in influenza HA (Wu et al., 2018; Wu et al., 2020; Wu et al., 2017). Unlike saturation mutagenesis in conventional deep mutational scanning, combinatorial mutagenesis enables us to measure the fitness of high-order mutants. By dissecting the mechanistic basis of epistasis, these studies have provided important insight into the biophysical constraints on HA antigenic evolution (Wu et al., 2018; Wu et al., 2020).

In this study, we aim to understand how epistatic effects influence NA antigenic evolution and investigate the underlying biophysical constraints. Specifically, we coupled combinatorial mutagenesis and next-generation sequencing to characterize the local fitness landscape of an NA antigenic region in six different human H3N2 strains that were isolated ~10 years apart. Our results indicate that the local fitness landscape of this NA antigenic region is highly correlated across six different genetic backgrounds. In-depth analyses further demonstrate that local net charge balancing is a biophysical constraint that governs the epistasis within this NA antigenic region. Lastly, we show that epistasis is correlated with residue coevolution in naturally circulating influenza strains.

## Results

### Local fitness landscape of an antigenic region on NA

**Figure 1.**
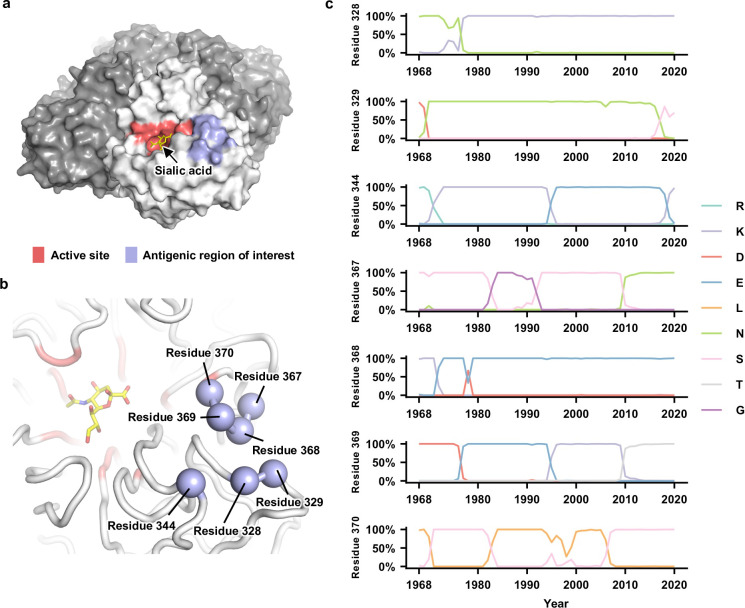
Natural evolution of an antigenic region in human H3N2 neuraminidase (NA). (**a**) The enzyme active site and the antigenic region of interest are highlighted as red and blue, respectively, on one protomer of the NA tetramer. The sialic acid within the active site is shown in yellow. (**b**) Seven residues in the antigenic region of interest are highlighted in blue spheres. (**c**) Natural occurrence frequencies of the amino acid variants that have a natural occurrence at >50% in any given year at the residues of interest are shown (Figure 1—source data 1 and Figure 1—source data 2). Of note, only those amino acid variants with a natural occurrence at >80% in any given year were included in our mutant libraries. Therefore, although D368 is shown in this plot, it was not included in our mutant libraries since it only reached a maximum occurrence of 67%. Figure 1—source data 1.Human H3N2 neuraminidase (NA) protein sequences. Figure 1—source data 2.Natural occurrence frequencies of the major amino acid variants at the residues of interest in different years.

When the structure of N2 NA was first reported in 1983 (Varghese et al., 1983), seven regions (I–VII) were proposed to be targeted by antibodies (Colman et al., 1983). Within regions I–III, mutations at residues 329, 344, 368, and 370 have been shown to escape monoclonal antibodies (Gulati et al., 2002; Varghese et al., 1988; Air et al., 1985). These four residues, along with residues 328 (region I), 367 (region III), and 369 (region III), form a cluster of seven residues that are very close in space (Figure 1a and b). Both residues 328 and 367 are under positive selection in human H3N2 NA (Westgeest et al., 2012), whereas coevolution of residues 367 and 369 in human H3N2 NA has created an N-glycosylation site (NXT) at residue 367 during 2010 (Figure 1c). These observations indicate that residues 328, 367, and 369 also participated in the antigenic drift of human H3N2 NA. By focusing on this seven-residue antigenic region, this study aimed to dissect the biophysical constraints on NA antigenic evolution.

**Figure 1—figure supplement 1.**
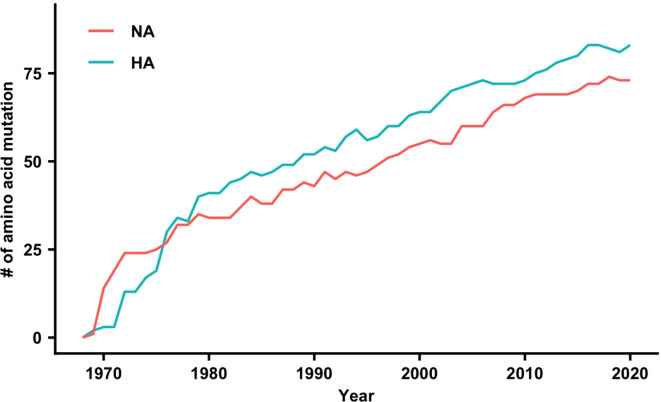
Accumulation of amino acid mutations in the hemagglutinin (HA) and neuraminidase (NA) of human H3N2 viruses. The number of amino acid mutations in the HA and NA relative to the ancestral strain, A/Hong Kong/1/1968 (HK68), is shown as a line graph. Consensus sequence of all isolates from a given year is used for calculating the number of mutations relative to HK68.

**Figure 1—figure supplement 2:**
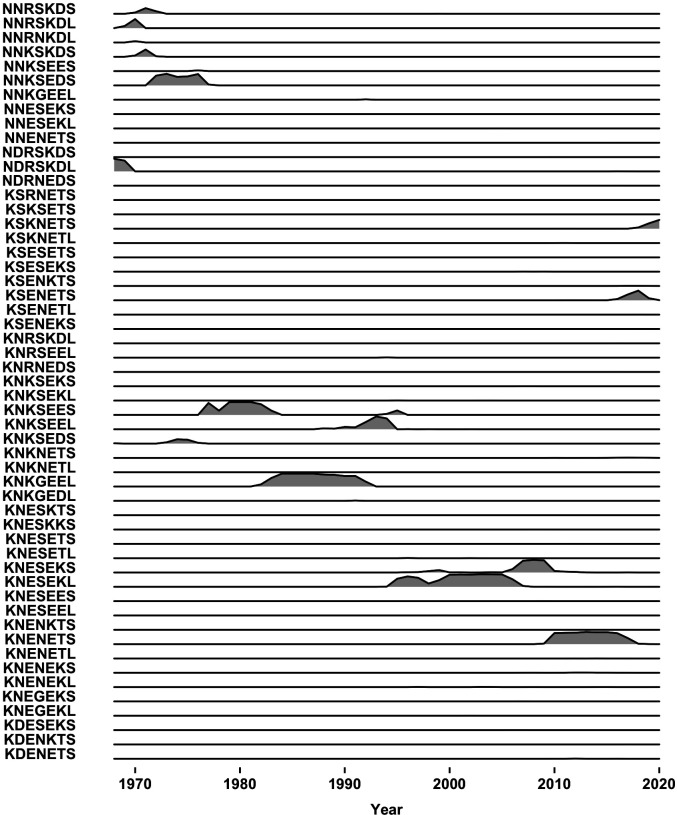
The frequency of haplotypes at the neuraminidase (NA) antigenic region of interest in circulating human H3N2 strains. In naturally circulating human H3N2 strains, 53 of the 864 haplotypes of interest were observed. The sequences of these 53 haplotypes are shown on the y-axis. Their occurrence frequency from 1968 to 2020 is plotted. For a frequency of 100%, the density curve would almost touch the next higher baseline. Some haplotypes have a very low occurrence frequency and may look like a complete flat line in the frequency diagram.

**Figure 1—figure supplement 3.**
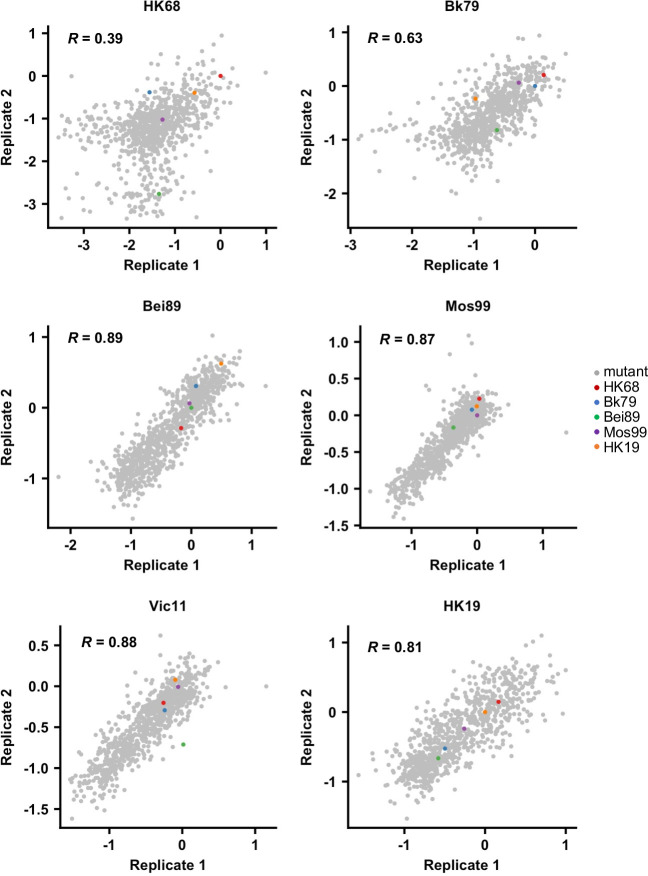
Correlations of fitness measurements between replicates. Correlations of variant fitness measurements between replicates of deep mutational scanning experiments are shown as scatterplots. Each data point within a scatterplot represents a unique variant. The Pearson correlation (R) between replicates is indicated. Data points corresponding to the wild type (WT) sequences of HK68, Bk79, Bei89, Mos99, and HK19 are colored as indicated. Of note, the WT sequence of Vic11 contains a naturally rare variant T329 that was not included in our study. Therefore, the WT sequence of Vic11 is not included in our mutant libraries.

We first compiled a list of amino acid variants at NA residues 328, 329, 344, 367, 368, 369, and 370 that reached an occurrence frequency of >80% in any given year during the natural evolution of human H3N2 viruses since 1968 (Figure 1c). This list includes two amino acid variants at residue 328 (Asn and Lys), three at residue 329 (Asn, Ser, and Asp), three at residue 344 (Lys, Glu, and Arg), three at residue 367 (Asn, Ser, and Gly), two at residue 368 (Lys and Glu), four at residue 369 (Lys, Glu, Asp, and Thr), and two at residue 370 (Leu and Ser). Together, there are 2 × 3 × 3 × 3 × 2 × 4 × 2 = 864 possible amino acid combinations (also called haplotypes) across these seven residues, although only 53 of them have been observed in naturally circulating human H3N2 strains (Figure 1—figure supplement 2). Using PCR primers that carry degenerate nucleotides (see Materials and methods and Supplementary file 1), these 864 variants were introduced into the NA of six different strains (genetic backgrounds) from 1968 to 2019, namely, A/Hong Kong/1/1968 (HK68), A/Bangkok/1/1979 (Bk79), A/Beijing/353/1989 (Bei89), A/Moscow/10/1999 (Mos99), A/Victoria/361/2011 (Vic11), and A/Hong Kong/2671/2019 (HK19). All these six strains were historical vaccine strains and isolated approximately 10 years apart.

To measure the virus replication fitness of all 864 variants in the six different genetic backgrounds of interest (i.e., HK68, Bk79, Bei89, Mos99, Vic11, and HK19), we employed a high-throughput experimental approach that coupled combinatorial mutagenesis and next-generation sequencing. Unlike conventional deep mutational scanning, which studies all possible single amino acid mutations across a protein or domain of interest, our approach analyzes all possible combinations of a subset of mutations. Subsequently, six different local fitness landscapes, each with 864 variants, were obtained. Fitness value of each variant, which was defined in log scale (see Materials and methods), was normalized to the corresponding wild type (WT), such that the WT fitness value was 0, whereas positive and negative fitness values represented beneficial and deleterious variants, respectively. Two biological replicates of each experiment were performed. Overall, a high correlation was observed between the replicates (Pearson correlation = 0.81–0.89) except for HK68 and Bk79 (0.39 and 0.63, respectively), mostly due to the high measurement noise for low fitness variants (Figure 1—figure supplement 3).

### Dynamics of the local fitness landscape across genetic backgrounds

**Figure 2.**
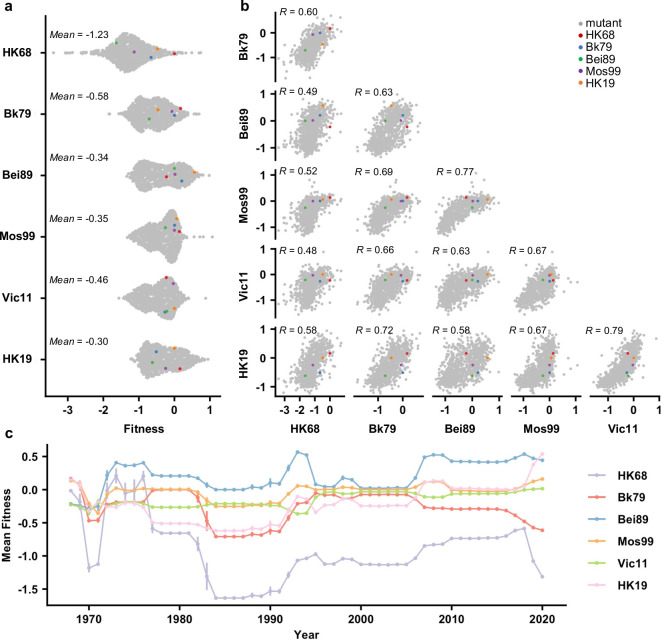
Comparing the local fitness landscapes of the neuraminidase (NA) antigenic region in six human H3N2 strains. (**a**) Variant fitness distributions in different genetic backgrounds are shown as a sina plot (Figure 2—source data 1). Each data point represents one variant. A total of 864 data points are present for each row (each genetic background). (**b**) Correlations of variant fitness distribution among different genetic backgrounds are shown, with each data point representing one variant. Pearson correlation coefficients (R) are indicated. (**a, b**) Data points corresponding to the wild type (WT) sequences of HK68, Bk79, Bei89, Mos99, and HK19 are colored as indicated. Of note, the WT sequence of Vic11 contains a naturally rare variant T329. Therefore, the WT sequence of Vic11 was not included in our mutant libraries. (**c**) Naturally occurring variants were grouped by the year of isolation, and their mean fitness in different genetic backgrounds is shown (Figure 2—source data 2). Different genetic backgrounds are represented by different lines, which are color coded as indicated on the right. Error bars represent the standard error of mean. This analysis included 66,562 NA sequences from human H3N2 strains that were isolated between 1968 and 2020 (Figure 1—source data 1). Figure 2—source data 1.Fitness value of each variant across six different genetic background. Figure 2—source data 2.The yearly mean fitness of naturally occurring variants in different genetic backgrounds.

To examine whether the local fitness landscape of the NA antigenic region of interest changes over time, fitness measurements of the 864 variants were compared across different genetic backgrounds (Figure 2a and b). Interestingly, while most variants were strongly deleterious (i.e., had a fitness of <-1) in HK68, variants with a fitness of <-1 were rare in other genetic backgrounds (Figure 2a). Notably, the difference in variant fitness distribution across genetic backgrounds was not due to the difference in their WT replication fitness since the viral titers from a rescue experiment were similar among HK68 WT, Bei89 WT, and Mos99 WT (Figure 2—figure supplement 1). In contrast, the fitness of individual variants correlated well across genetic backgrounds (Pearson correlation = 0.48–0.79, Figure 2b), despite their differences in variant absolute fitness values (Figure 2a). These results demonstrate that although epistasis exists between the seven-residue antigenic region and the rest of the NA sequence, such epistasis is largely variant-nonspecific. Consequently, the topology of the local fitness landscape is highly conserved across genetic backgrounds. This observation is very different from a similar study on a major antigenic site of HA, where the topology of the local fitness landscape differed dramatically among genetic backgrounds (Wu et al., 2020).

**Figure 2—figure supplement 1.**
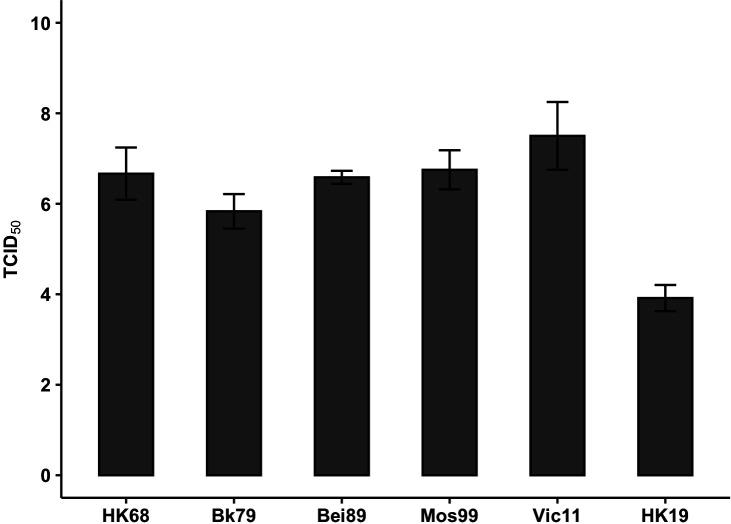
Virus rescue experiment of wild type (WT) strains. The titer of six WT strains (HK68, Bk79, Bei89, Mos99, Vic11, and HK19) from a virus rescue experiment is shown. Error bar represents standard error from three biological replicates. This rescue experiment was performed using the six internal segments from A/WSN/33 (H1N1) and hemagglutinin (HA) segment from HK68.

We further examined the fitness of naturally occurring variants in different genetic backgrounds (HK68, Bk79, Bei89, Mos99, Vic11, and HK19) (Figure 2c). Most natural variants have a fitness between –0.5 and 0.5 on the background of Bk79, Bei89, Mos99, Vic11, and HK19. In contrast, most natural variants from 1980s onward are strongly deleterious with fitness <-1 on the background of HK68, indicating many natural variants would not have emerged if the genetic background had not evolved. In other words, the emergence of natural variants in the antigenic region of interest is contingent on the evolution of the rest of the NA sequence. This result is consistent with the observation that HK68 has a much lower mutational tolerance at this antigenic region (Figure 2a).

### Conservation of pairwise epistasis across genetic backgrounds

**Figure 3.**
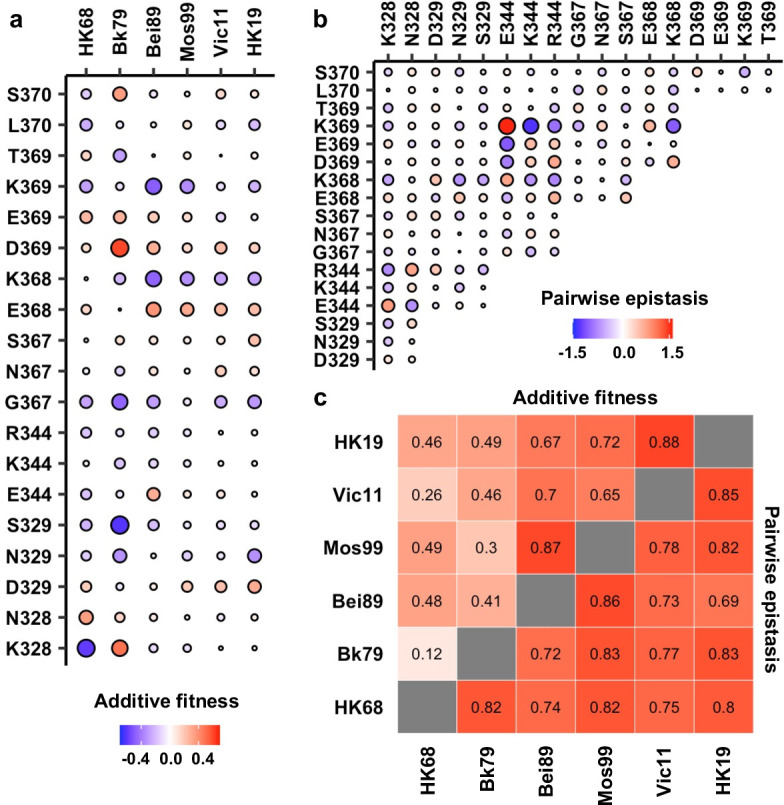
Inference of additive fitness and pairwise epistasis. (**a**) Parameters for additive fitness in different genetic backgrounds are shown. (**b**) Pairwise epistatic effects show strong correlations among six different genetic backgrounds, and therefore, Bk79 is shown as a representative. The identity of a given double amino acid variant is represented by the labels on the x- and y-axes. The same plots for other genetic backgrounds are shown in Figure 3—figure supplement 5. (**a, b**) Positive additive fitness and pairwise epistasis are in red. Negative additive fitness and pairwise epistasis are in blue. The magnitude is proportional to the size of the circle. (**c**) Correlation matrices of additive fitness and pairwise epistasis among six genetic backgrounds are shown as a heatmap. See Figure 3—figure supplements 4 and 5 for the related scatter plots.

To understand the biophysical constraints on NA antigenic evolution, the local fitness landscapes were decomposed into additive fitness effects of individual amino acid variants and pairwise epistasis between amino acid variants (Starr and Thornton, 2016). Additive fitness describes the independent contributions of each amino acid variant to fitness, whereas pairwise epistasis describes the nonadditive interactions between amino acid variants. For each genetic background, additive fitness and pairwise epistasis were inferred from the variant fitness data using an established statistical learning model (Tareen et al., 2020) (see Materials and methods). The model was evaluated using repeated k-fold cross-validation and hyperparameters were chosen by maximizing the R^2^ of model prediction and the Pearson correlation coefficient of model parameters (Figure 3—figure supplement 1). While the correlations between additive fitness contributions varied hugely across genetic backgrounds (Pearson correlation = 0.12–0.88, Figure 3a and c, Figure 3—figure supplement 2), we observed generally strong correlations between pairwise epistatic effects across the six different genetic backgrounds (Pearson correlation = 0.69–0.86, Figure 3b and c, Figure 3—figure supplement 3). Of note, the variation of additive fitness contributions across genetic backgrounds does not seem to strictly depend on the similarity between genetic backgrounds since the correlation between the additive fitness contributions of HK68 and HK19 (Pearson correlation = 0.46) is much higher than that of HK68 and Bk79 (Pearson correlation = 0.12), which have a shorter time separation. This observation points at a possibility for complex epistatic interactions between the antigenic region of interest and other regions on NA. Overall, these results indicate that pairwise epistasis, but not additive fitness, is highly conserved at the NA antigenic region of interest across genetic backgrounds. Consistently, an ‘additive-only’ model, without accounting for epistasis, shows a poor fit to the fitness landscape data compared to the model above with epistasis (Figure 3—figure supplement 4a), despite the fact that the inferred additive fitness effects correlate well between the two models (Figure 3—figure supplement 4b).

**Figure 3—figure supplement 1.**
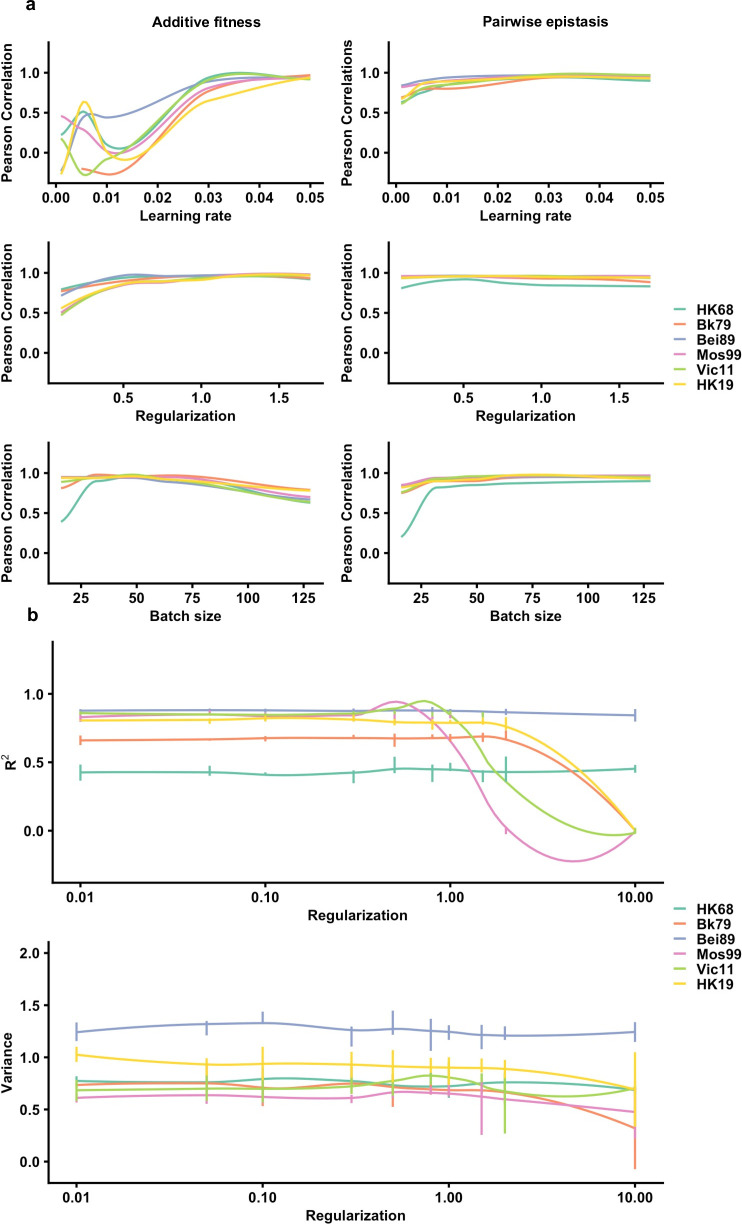
Evaluation of model hyperparameters using repeated k-fold cross-validation. (**a**) The relationship between different hyperparameters (learning rate, regularization, and batch size) and model robustness is evaluated. (**b**) The relationship between different regularization hyperparameter and model fit is evaluated.

**Figure 3—figure supplement 2.**
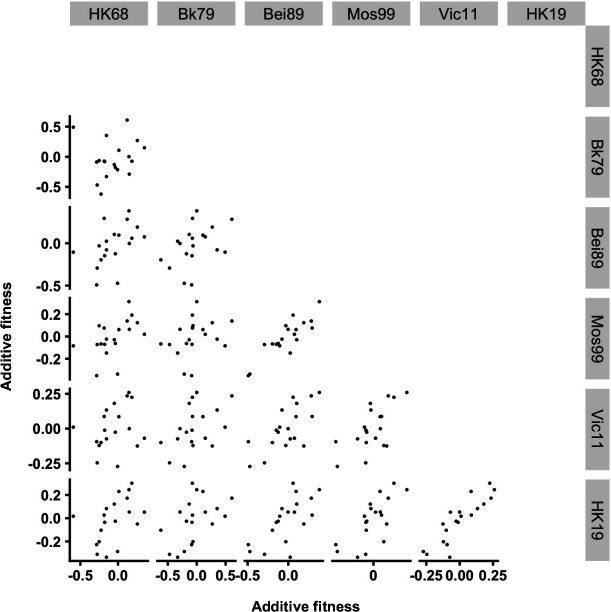
Correlations of additive fitness among genetic backgrounds. The 19 parameters for additive fitness are compared among genetic backgrounds, with each data point representing one parameter. The correlations of additive fitness among genetic backgrounds varied hugely (Pearson correlation = 0.12–0.88).

**Figure 3—figure supplement 3.**
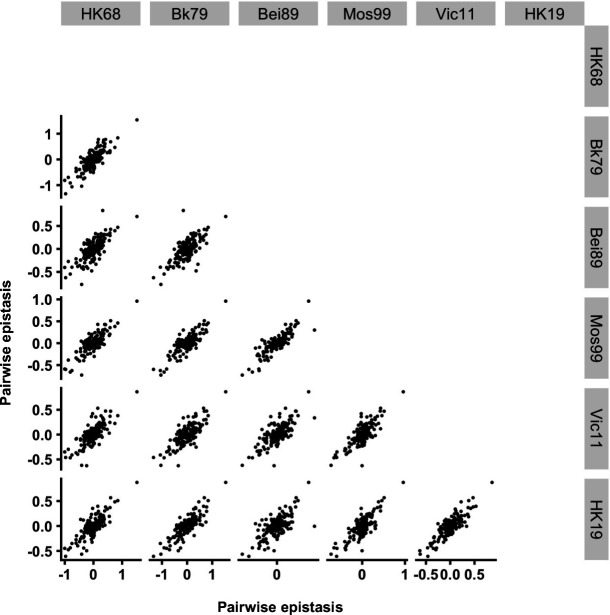
Correlations of pairwise epistasis among genetic backgrounds. The 153 parameters for pairwise epistasis are compared among genetic backgrounds, with each data point representing one parameter. The correlations of pairwise epistasis among genetic backgrounds are generally strong (Pearson correlation = 0.69–0.86).

**Figure 3—figure supplement 4.**
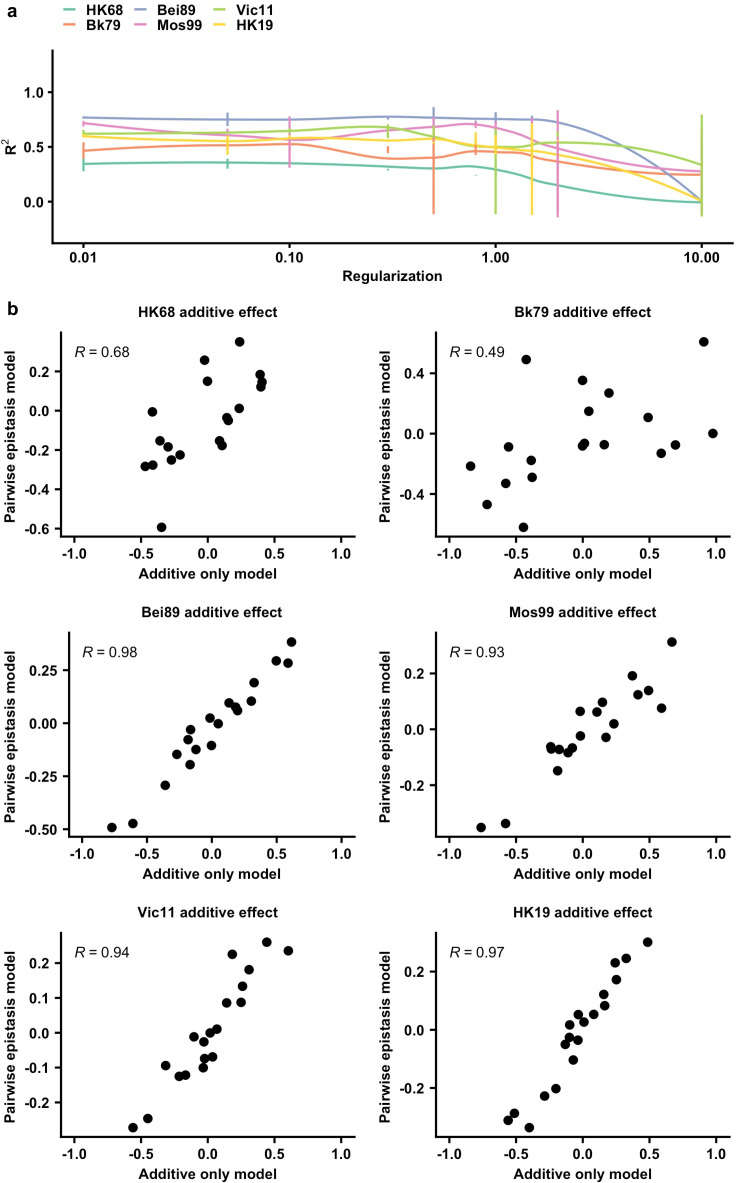
Evaluation of additive-only model. (**a**) The relationship between different regularization hyperparameter and model fit is evaluated. The best R^2^ for the additive-only model are 0.38 (HK68), 0.51 (Bk79), 0.8 (Bei89), 0.71 (Mos99), 0.65 (Vic11), and 0.6 (HK19), and the best R^2^ for the pairwise model are 0.48 (HK68), 0.7 (Bk79), 0.88 (Bei89), 0.86 (Mos99), 0.86 (Vic11), and 0.82 (HK19), respectively. (**b**) The inferred additive fitness effects correlation between two models. Pearson correlation is indicated.

**Figure 3—figure supplement 5.**
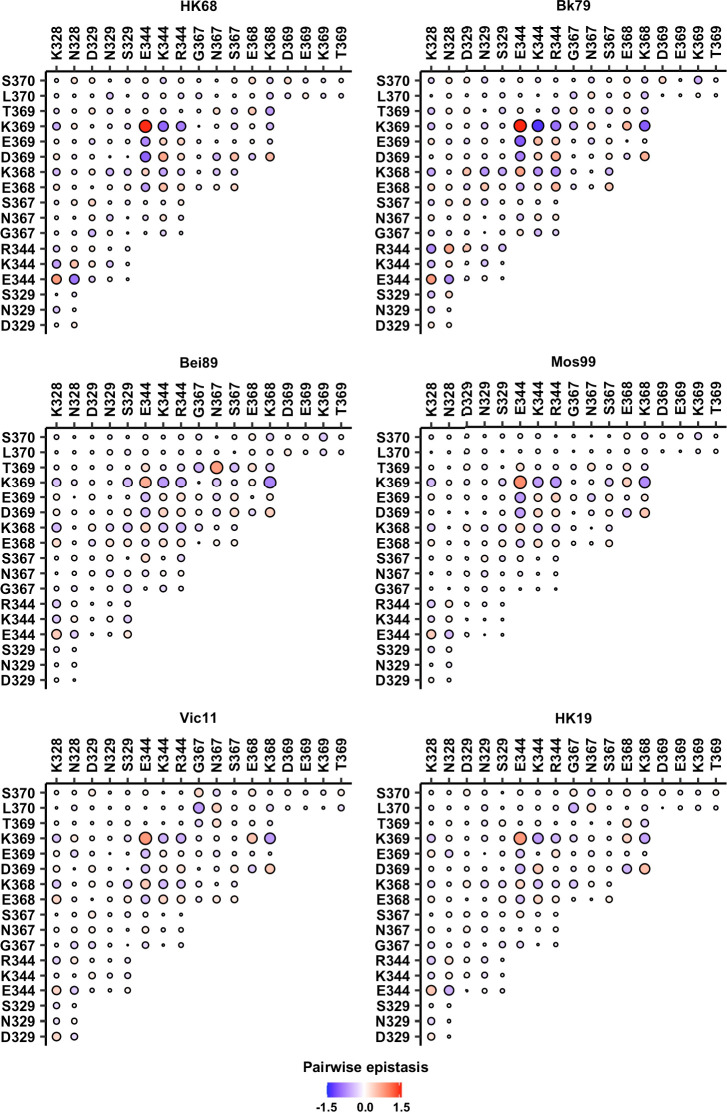
Pairwise epistasis in different genetic backgrounds. Positive epistasis is in red, while negative epistasis is in blue. The magnitude of epistasis is proportional to the size of the circle. The identity of a given double amino acid variant is represented by the labels on the x- and y-axes.

### Local net charge imposes a biophysical constraint on NA antigenic evolution

**Figure 4.**
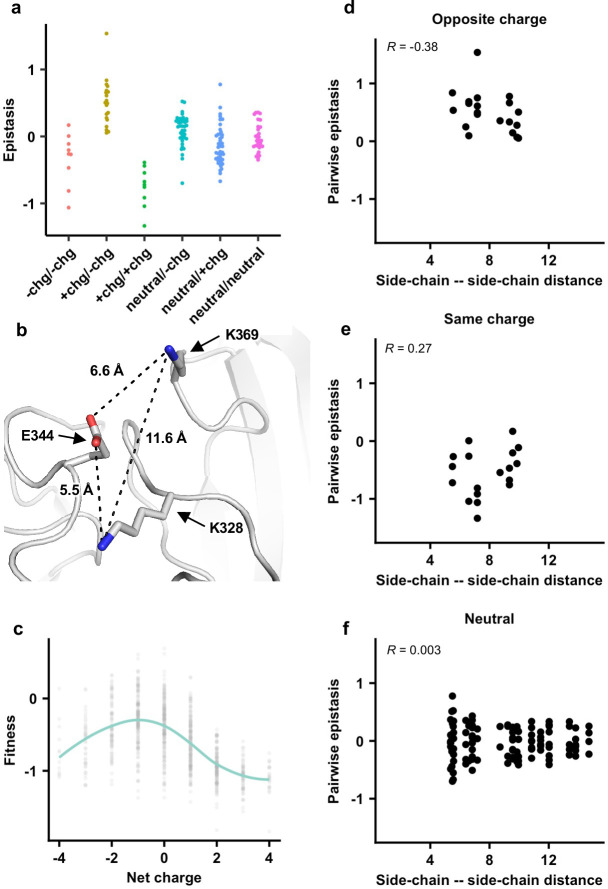
The importance of local net charge in the neuraminidase (NA) antigenic region. (**a**) Pairwise epistasis in genetic background Bk79 is shown and classified based on amino acid charges. +chg represents positively charged amino acids (K/R), -chg represents negatively charged amino acids (D/E), and neutral represents the remaining amino acids. The same plots for other genetic backgrounds are shown in Figure 4—figure supplement 1. (**b**) NA structure from H3N2 A/Memphis/31/98 (PDB 2AEP), which has K328, E344, and K369, was analyzed (Gulati et al., 2002). A similar NA structure from H3N2 A/Tanzania/205/2010, which has K328 and E344, is shown in Figure 4—figure supplement 2. (**c**) The relationship between variant fitness and net charge in genetic background Bk79 is shown. A smooth curve was fitted by loess and shown in teal. The same plots for other genetic backgrounds are shown in Figure 4—figure supplement 4. (**d–f**) Relationship between the side-chain–side-chain distances (Figure 4—source data 1) and epistasis for (**d**) variant pairs with opposite charges, (**e**) variant pairs with the same charge, and (**f**) variant pairs that involve a neutral amino acid in genetic background Bk79 is shown. The same plots for other genetic backgrounds are shown in Figure 4—figure supplement 3. Pearson correlation coefficient (R) is indicated. Figure 4—source data 1.Pairwise side-chain–side-chain distances within neuraminidase (NA) antigenic region.

Next, we investigated the biophysical constraints that have led to such conserved patterns of epistatic interactions in the NA antigenic region of interest. We noticed that amino acid variants with opposite charges usually exhibited positive epistasis, whereas amino acid variants with the same charge usually exhibited negative epistasis (Figures 3b and 4a, Figure 3—figure supplement 5, Figure 4—figure supplement 1; see Materials and methods). In contrast, pairwise interactions that involved a neutral amino acid variant did not show any bias towards positive or negative epistasis. These results suggest that balancing of charged amino acid variants imposes a key biophysical constraint on the evolution of this NA antigenic region.

**Figure 4—figure supplement 1.**
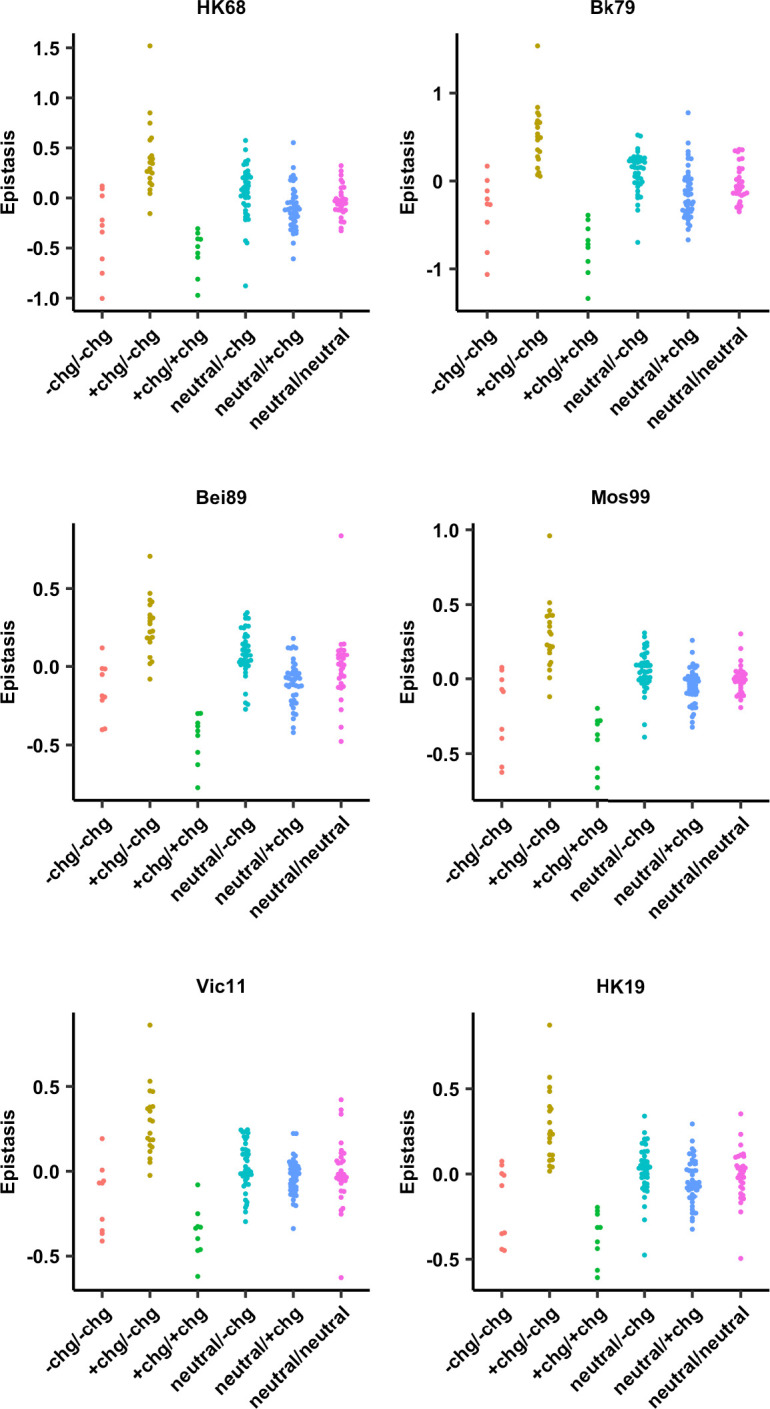
Pairwise epistasis was classified based on the side-chain charge in each pair of amino acid variants. +chg represents positively charged amino acids (K/R), -chg represents negatively charged amino acids (D/E), and neutral charge represents the remaining amino acids.

**Figure 4—figure supplement 2.**
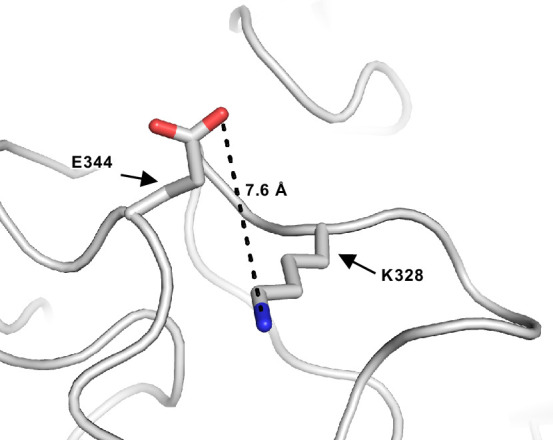
Neuraminidase (NA) structure from H3N2 A/Tanzania/205/2010 (PDB 4GZO) (Zhu et al., 2012), which has K328 and E344, is shown. The distance between the side-chain carboxylate oxygen of E344 and the side-chain amine nitrogen of K328 is indicated.

**Figure 4—figure supplement 3.**
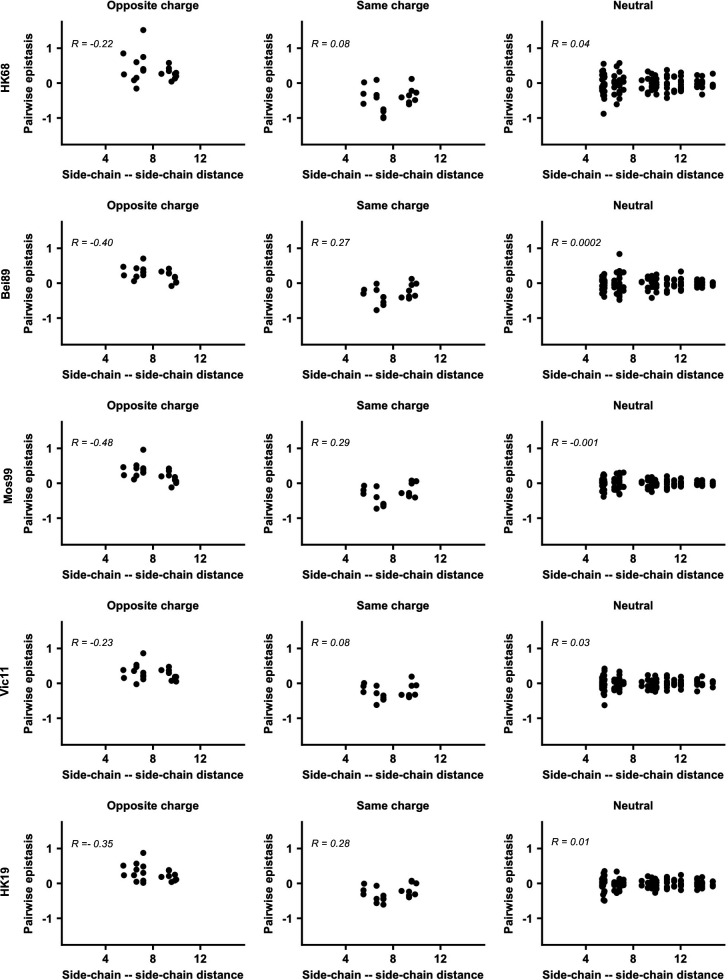
Relationship between the side-chain–side-chain distance and pairwise epistasis. The side-chain–side-chain distance was computed based on the geometric center of the side-chain atoms from each residue of interest (Hockenberry and Wilke, 2019).

**Figure 4—figure supplement 4.**
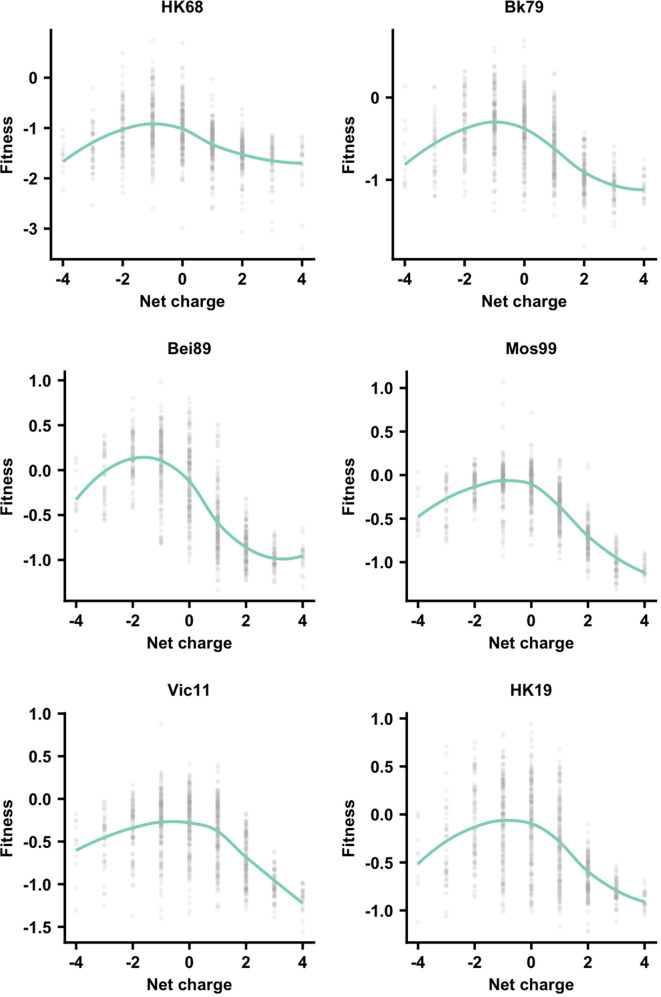
Relationship between variant fitness and the local net charge. The relationship between variant fitness and the local net charge in the indicated genetic background is shown. A smooth curve was fitted by loess and shown in teal.

To further probe the mechanism of epistasis in this NA antigenic region, we analyzed a published crystal structure of human H3N2 NA that has K328, E344, and K369 (Figure 4b; Venkatramani et al., 2006). Both variant pairs E344/K369 and K328/E344 exhibited positive epistasis across all genetic backgrounds, whereas K328/K369 exhibited negative epistasis (Figure 3—figure supplement 5). In fact, E344 and K369 had the strongest positive epistasis in five of the six genetic backgrounds of interest (except Bei89). Our structural analysis showed that the distance between the side-chain carboxylate oxygen of E344 and the side-chain amine nitrogen of K369 is 6.6 Å, whereas the distance between the side-chain carboxylate oxygen of E344 and the side-chain amine nitrogen of K328 is 5.5 Å (Figure 4b, Figure 4—figure supplement 2). At these distances, salt bridges cannot be formed and the electrostatic attraction force is negligible (Kumar and Nussinov, 2002; Shashikala et al., 2019). Similarly, the distance between the side-chain amine nitrogen atoms of K328 and K369 is 11.6 Å (Figure 4b), which is too far for any significant electrostatic repulsion force (Shashikala et al., 2019). As a result, direct side-chain–side-chain interaction via electrostatic attraction or repulsion is unlikely to be a major determinant for epistasis in this NA antigenic region. Consistently, pairwise epistasis between two amino acid variants does not correlate with their side-chain–side-chain distances (Figure 4d–f, Figure 4—figure supplement 3), further substantiating that direct side-chain–side-chain interaction is not a determinant for epistasis here.

We then analyzed the relationship between variant fitness and local net charge as a molecular phenotype. Here, local net charge was defined as the sum of charges at the seven residues of interest (residues 328, 329, 344, 367, 368, 369, and 370), where positively charged amino acids (K and R) were +1 and negatively charged residues (D and E) –1. In all genetic backgrounds, variants tended to have a higher fitness when the local net charge was around –1, while variants with a more positive or negative local net charge usually had a lower fitness (Figure 4c, Figure 4—figure supplement 4). Overall, our results demonstrate that the local net charge is a key molecular phenotype with a biophysical function that is under balancing selection. This biophysical phenotype imposes a strong constraint on the evolution of the NA antigenic region, reflected in the conserved epistatic interactions between amino acid variants of this region.

### Impact of local net charge and epistasis on NA evolution

**Figure 5.**
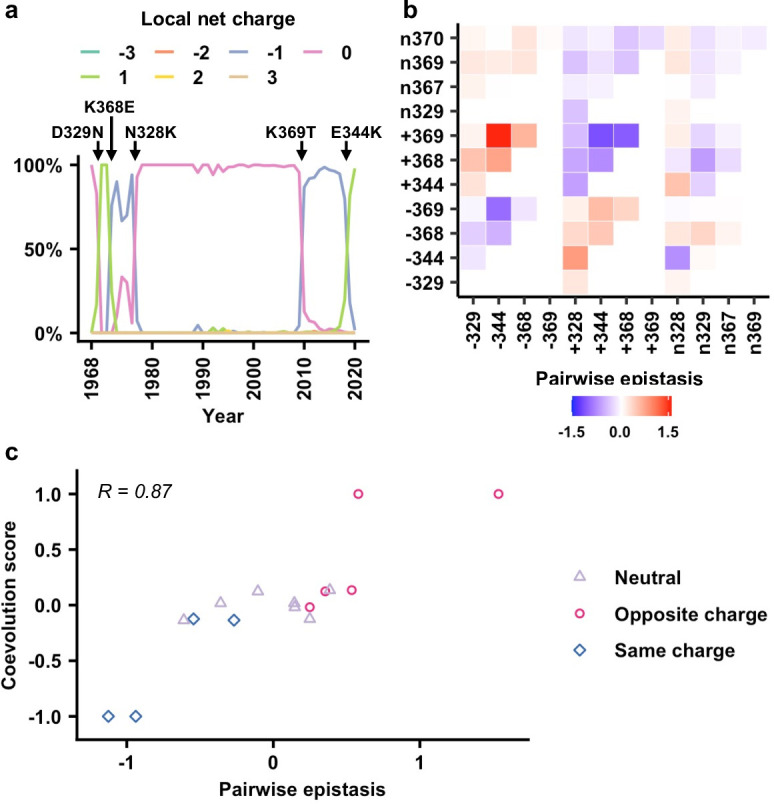
Correlation between epistasis and coevolution of charge states in the neuraminidase (NA) antigenic region. (**a**) The natural evolution of frequency of haplotypes by local net charge at the NA antigenic region of interest is shown (Figure 5—source data 1). Key mutation events that led to changes in local net charge are indicated. (**b**) Pairwise epistasis of charge states among different residues. Amino acids were classified based on charges. (+) represents positively charged amino acids (K/R). (-) represents negatively charged amino acids (D/E). (n) represents the remaining amino acids. (**c**) Relationship between coevolution score (Figure 5—source data 2) and pairwise epistasis in genetic background Bk79 is shown. Pearson correlation coefficient (R) is indicated. The same plots for other genetic backgrounds are shown in Figure 5—figure supplement 4. Figure 5—source data 1.The local net charge at the neuraminidase (NA) antigenic region of interest in different strains from 1968 to 2020. Figure 5—source data 2.Pairwise coevolution score for pairs of charge states that emerge or disappear within 5 years.

Next, we aimed to understand whether the local net charge at the NA antigenic region of interest influences its evolution in circulating human H3N2 strains. We retrieved 66,562 human H3N2 NA sequences spanning from 1968 to 2020 from the Global Initiative for Sharing Avian Influenza Data (GISAID) (Shu and McCauley, 2017). Most natural variants in the antigenic region of interest had a local net charge between –1 and +1 (Figure 5a). Natural variants with a local net charge of −3,–2, + 2, or +3 could also be observed but were rare. This observation suggests that the natural evolution of this NA antigenic region is constrained by balancing the local net charge.

**Figure 5—figure supplement 1.**
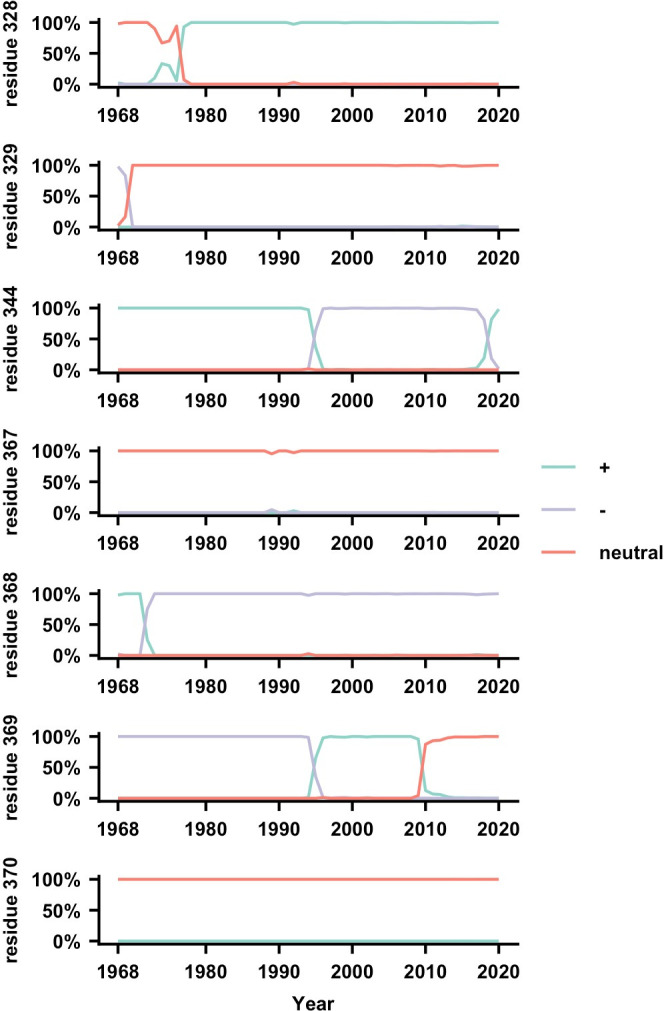
The natural occurrence frequencies of the major amino acid charge states are shown. (+) represents positively charged amino acids (K/R), (-) represents negatively charged amino acids (D/E), and (n) represents the remaining amino acids.

**Figure 5—figure supplement 2.**
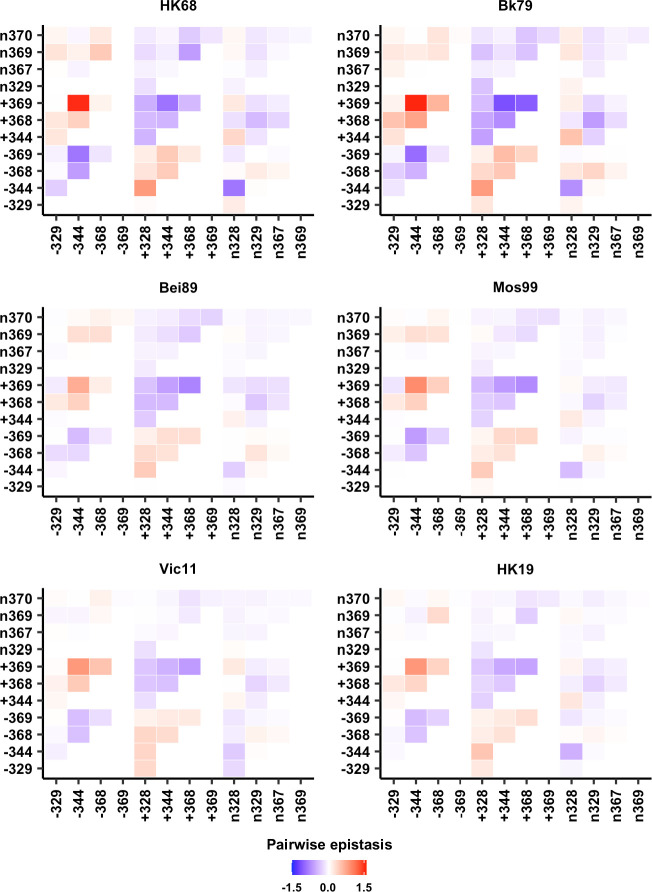
Pairwise epistasis of charged states among seven residues. Amino acids were classified based on their charge state. (+) represents positively charged amino acids (K/R), (-) represents negatively charged amino acids (D/E), and (n) represents the remaining amino acids. For instance, –344 represents negatively charged amino acids (D/E) at residue 344. The epistasis of +344/–369 would then be the average among that of K344/D369, R344/D369, K344/E369, and R344/E369.

**Figure 5—figure supplement 3.**
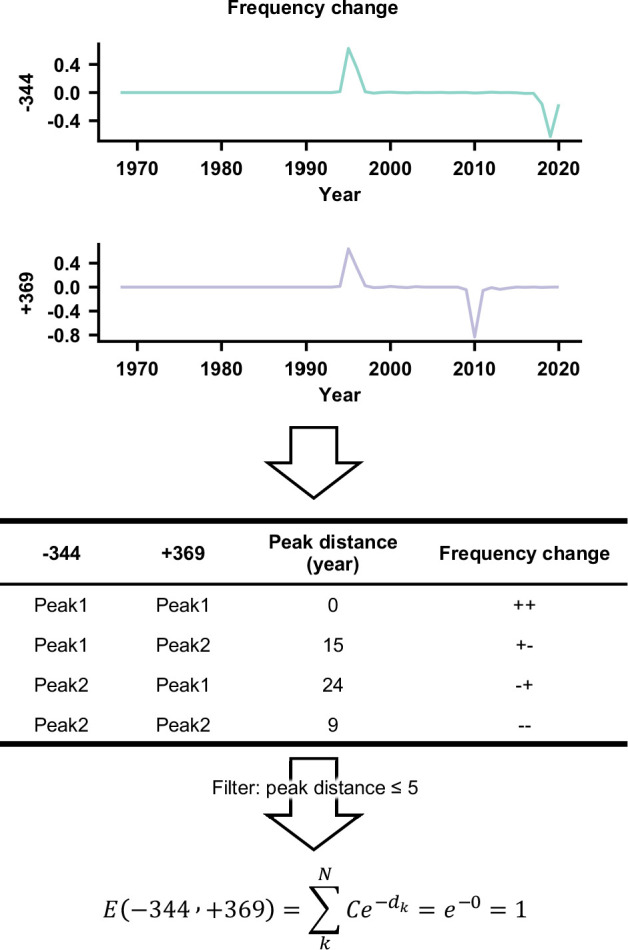
Schematic overview of calculating the coevolution score. The natural frequency changes of amino acids charges at residues 328, 329, 344, 367, 368, 369 and 370 from year to year were calculated. In this simplified example here, one pair of charge states (–344/+369) was used for demonstration. –344 represents negatively charged amino acids (D/E) at residue 344 and +369 means positively charged amino acids (K/R) at residue 369. The frequency change represents frequency in year n minus frequency in year n–1. Both –344 and +369 have two peaks that are above our cutoff of 5% frequency change. Subsequently, peaks from different residues were compared and the distance between each pair of peaks was calculated. Each pair of peaks was classified based on their signs. For example, ‘++’ represents both peaks are positive in magnitude. Eventually, coevolution score of a given pair of charge states was calculated as the sum of exponential decay function of all pairwise peak distances. C = 1 if both peaks have the same sign (++ or --), otherwise C = –1 (+- or -+). In this study, pairs of peaks with a time separation $d_{k}$ of larger than 5 years were discarded.

**Figure 5—figure supplement 4.**
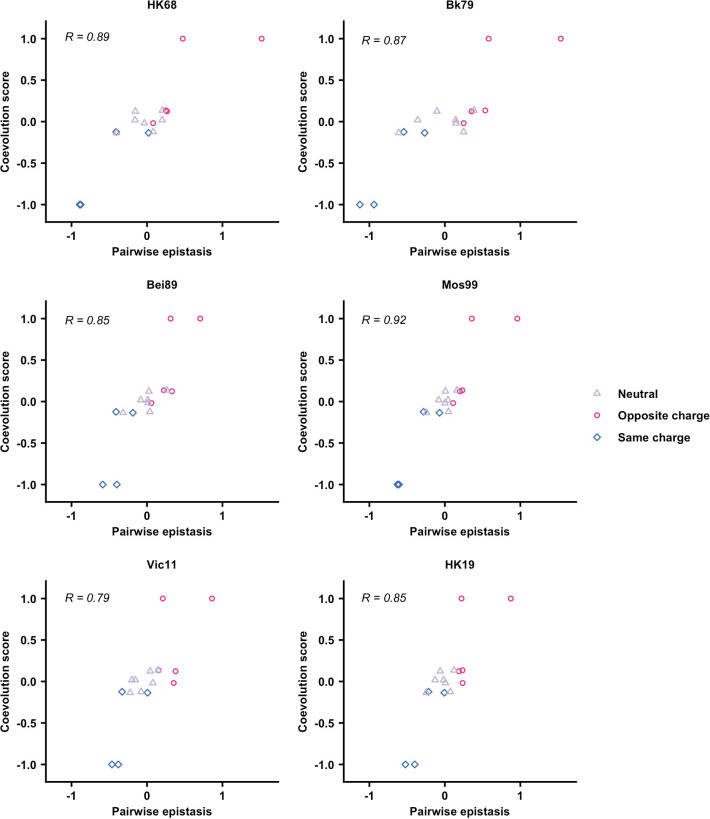
Correlations between coevolution score and pairwise epistasis in different genetic backgrounds. The relationship between the coevolution score and pairwise epistasis of charge states in the indicated genetic background is shown. Pearson correlation is indicated.

**Figure 5—figure supplement 5.**
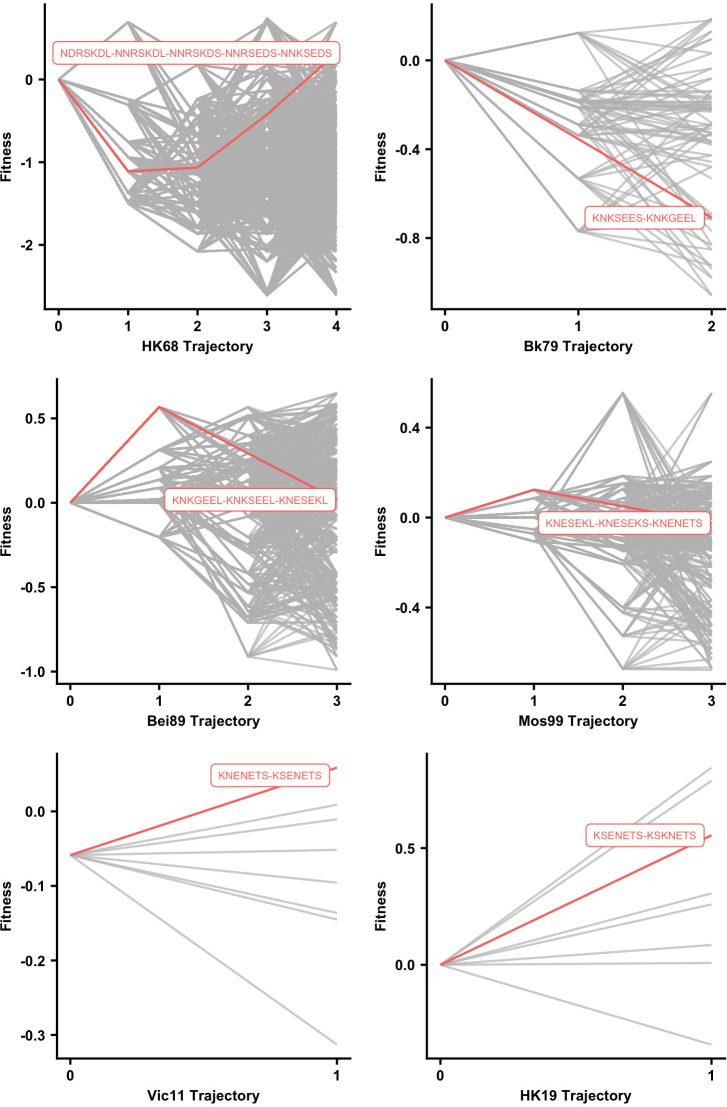
An ensemble of evolutionary trajectories constructed from fitness data. Evolutionary trajectories were constructed based on the fitness data (see Materials and methods). The realized trajectory in nature is highlighted in red. This result demonstrates that natural evolutionary trajectory does not always maximize the replication fitness. This discrepancy is likely due to other selection pressures in nature (e.g., immune selection pressure) that were not being captured in our in vitro fitness measurement or due to changes in the genetic background through epistatic interactions that were not captured by our experiments.

**Figure 5—figure supplement 6.**
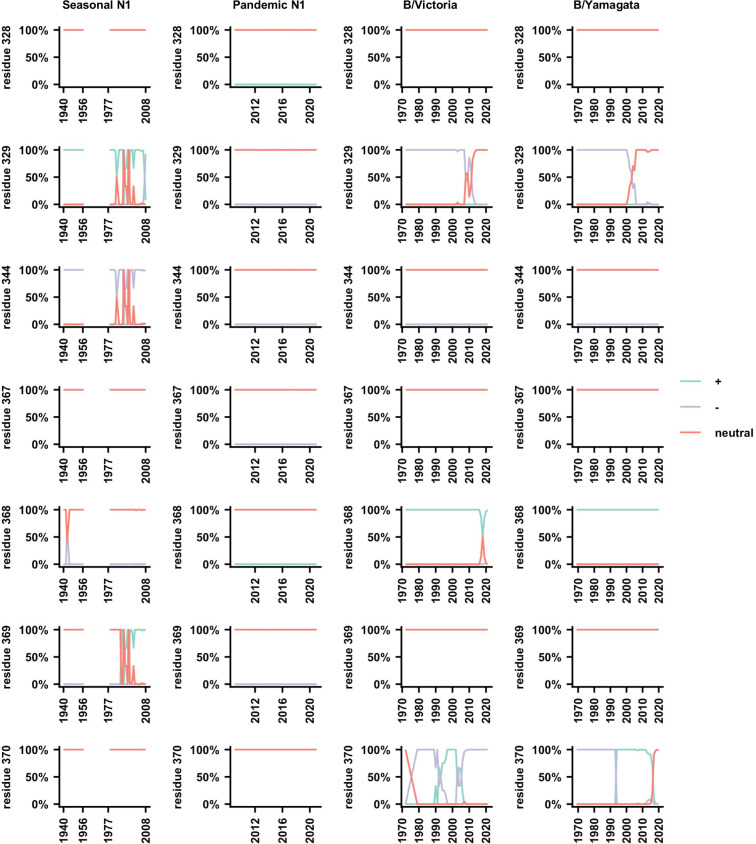
The natural occurrence frequencies of the charge states in the homologous regions of H1N1 and influenza B are shown. (+) represents positively charged amino acids (K/R), (-) represents negatively charged amino acids (D/E), and (n) represents the remaining amino acids. N2 numbering is used in this analysis.

We further tested whether epistasis is correlated with the evolution of local net charge at the NA antigenic region of interest. A coarse-grained analysis that only considered three charged states, namely, positive, negative, and neutral, for each residue was performed (see Materials and methods, Figure 5—figure supplement 1). In this analysis, amino acids were classified into (+) as positively charged (amino acids K/R), (-) as negatively charged (amino acids D/E), and (n) as neutral representing the remaining amino acids. We then computed the pairwise epistasis between different charge classes in two loci by averaging over all the corresponding amino acids in each class (Figure 5b, Figure 5—figure supplement 2). For example, the epistasis between positive charge at residue 344 and negative charge at residue 369 (i.e., +344/–369) is the averaged epistasis over K344/D369, R344/D369, K344/E369, and R344/E369. In addition, we evaluated a coevolution score between a pair of charged states at two different residues (Materials and methods, Figure 5—figure supplement 3). In our definition, two charge states that emerged or disappeared shortly one after another would have a positive coevolution score. In contrast, emergence of a charge state in one residue followed by disappearance of a charge state in another residue would result in a negative coevolution score. Our analysis only included pairs of charge states that emerged or disappeared within 5 years from each other. Since residues 367 and 370 were dominated by neutral charge since 1968, they were not included on our coevolution analysis (Figure 5—figure supplement 1).

When we compared the coevolution score with the pairwise epistasis (Figure 5c, Figure 5—figure supplement 4), high correlation was observed (Pearson correlation = 0.79–0.92). Specifically, pairs with opposite charges usually have positive coevolution scores and positive epistasis, whereas pairs with the same charge usually have negative coevolution scores and negative epistasis. We also attempted to use the fitness data to predict the identity of amino acid mutations along the evolutionary trajectory. Nonetheless, the realized evolutionary trajectories in nature are seldom the most probable ones among an ensemble of trajectories that were constructed from the fitness data (Figure 5—figure supplement 5). As a result, while our analysis demonstrates that a biophysically grounded epistatic landscape is correlated with the natural coevolution of residues in this NA antigenic region, additional considerations are needed for the prediction of the exact amino acid mutations along the evolutionary trajectory.

## Discussion

Understanding the biophysical constraints is a key to model the molecular evolution of proteins (Echave and Wilke, 2017; Luksza and Lässig, 2014; Nourmohammad et al., 2013; Otwinowski et al., 2018; Mustonen et al., 2008; Wylie and Shakhnovich, 2011; Lässig et al., 2017). Through a systematic analysis of pairwise epistasis, this study shows that the local net charge is a major biophysical molecular phenotype that constrains the evolution of an antigenic region in human influenza H3N2 NA. An important feature for this antigenic region of interest is that pairwise epistasis is highly conserved across diverse genetic backgrounds, which in turn is correlated with residue coevolution in naturally circulating human influenza H3N2 virus. Although the homologous regions in the H1N1 NA and influenza B NA do not evolve as extensively as human influenza H3N2 NA (Figure 5—figure supplement 6), residue 329 coevolves with 344 in seasonal influenza H1N1 NA, indicating that similar local net charge balancing may also influence the evolution of H1N1 NA. In fact, earlier theoretical studies have indicated that charge balancing is one of the strongest signatures of correlated evolution across different protein families (Neher, 1994; Magliery and Regan, 2004; Callahan et al., 2011). Our study here further provided empirical evidence for this phenomenon.

A key finding of this study is that the optimal local net charge at the antigenic region of interest is slightly negative, and an increase or decrease in local net charge is deleterious. This characteristic suggests that the local net charge may be the key molecular phenotype under stabilizing and balancing selection, imposing a biophysical constraint on evolution of the NA antigenic region. This local net charge may have several nonexclusive functional roles. First, since the host cell membrane and sialylated glycan receptor are both negatively charged, charge distribution on the virus surface, including the antigenic region of interest, may affect the kinetics of host membrane attachment and virus release. Second, the antigenic region of interest is proximal to the catalytic active site (Figure 1a), the local net charge may affect the catalytic efficiency of NA. Lastly, the local net charge may influence the protein stability of NA (de Graff et al., 2016; Raghunathan et al., 2013). Understanding the detailed molecular mechanisms of biophysical constraints, albeit beyond the scope of this study, will likely further enhance the ability to model evolution.

One interesting observation in this study is that the ancestral strain HK68 has a much lower mutational tolerance at the antigenic region of interest than the subsequent strains, although the topology of the local fitness landscapes is largely conserved across strains. This result suggests that other biophysical features of NA are evolving over time and that they epistatically interact with the antigenic region of interest in a residue nonspecific manner. Nonspecific epistasis is often related to protein stability (Starr and Thornton, 2016), which is best described by the threshold robustness model (Bloom et al., 2006). Under the threshold robustness model, slightly destabilizing mutations may have neutral fitness when the protein has an excess stability margin but are deleterious when the protein is marginally stable. Threshold robustness model can be used to explain the differential variant fitness distribution among the six genetic backgrounds in our study. For example, it is possible that Mos99 NA has an excess stability margin such that many variants are nearly neutral despite being slightly destabilizing. In contrast, the same slightly destabilizing variants are highly deleterious in HK68 NA because it is marginally stable. When comparing the variant fitness in different genetic backgrounds, some nonlinearity can be observed (Figure 2b), which is a feature of the threshold robustness model (Otwinowski et al., 2018; Bloom Jesse et al., 2005). Nevertheless, additional studies are needed to confirm whether the difference in variant fitness distribution among genetic backgrounds is due to protein stability or other biophysical factors.

Predicting the evolution of human influenza virus is a challenging task, yet important for seasonal influenza vaccine development. An accurate predictive model of human influenza evolution likely requires an integration of epitope information (Luksza and Lässig, 2014), antigenic data (Neher et al., 2016), mutant fitness measurement (Lee et al., 2018), and experimental selection for antibody escape variants (Li et al., 2016). This work further suggests that a biophysical epistatic model of antigenic fitness landscape can also be instrumental in modeling the evolution of human influenza virus. As the knowledge about the evolutionary biology of influenza virus accumulates, a unifying model that can accurately predict emerging mutation may one day be built.

## Materials and methods

**Key resources table:** 

Reagent type (species) or resource	Designation	Source or reference	Identifiers	Additional information
Cell line (*Homo sapiens*)	MDCK-SIAT1	Sigma-Aldrich	Cat#: 05071502-1VL	
Cell line (*H. sapiens*)	HEK 293T	Scripps Research	N/A	
Strain, strain background (*Escherichia coli*)	MegaX DH10B T1R	Thermo Fisher Scientific	Cat#: C640003	
Recombinant DNA reagent	A/WSN/33 (H1N1) eight-plasmid reverse genetics system	Neumann et al., 1999	N/A	
Commercial assay or kit	Lipofectamine 2000	Thermo Fisher Scientific	Cat#: 11668019	
Commercial assay or kit	KOD Hot Start DNA Polymerase	EMD Millipore	Cat#: 71086-3	
Software, algorithm	Python	Python Software Foundation	RRID:SCR_008394	
Software, algorithm	R	R Core Team	RRID:SCR_001905	

### Cell lines

HEK 293T (human embryonic kidney) cells and MDCK-SIAT1 (Madin–Darby canine kidney) cells were used in this study. The identification of the cell lines was confirmed morphologically. Cells were maintained in a humidified 37°C, 5% CO_2_ incubator and cultured in Dulbecco’s modified Eagle’s medium (DMEM) (Life Technologies), supplemented with 10% fetal bovine serum (FBS) (VWR), and 1% penicillin-streptomycin (Life Technologies). Cells were tested monthly for mycoplasma contamination. Mycoplasma contamination was not detected.

### Recombinant influenza virus

All H3N2 viruses generated in this study were based on the influenza A/WSN/33 (H1N1) eight-plasmid reverse genetics system (Neumann et al., 1999). Chimeric 6:2 reassortments were employed with the HA ectodomains from the H3N2 A/Hong Kong/1/1968 (HK68) and the entire NA coding region from the strains of interest (Wu et al., 2017). For HA, the ectodomain was from HK68, whereas the noncoding region, N-terminal secretion signal, C-terminal transmembrane domain, and cytoplasmic tail were from A/WSN/33. For NA, the entire coding region was from the strains of interest, whereas the noncoding region of NA was from A/WSN/33. H3N2 strains of interest in this study were as follows with GISAID (Shu and McCauley, 2017) accession numbers in parentheses: A/Hong Kong/1/1968 (EPI_ISL_245769), A/Bangkok/1/1979 (EPI_ISL_122020), A/Beijing/353/1989 (EPI_ISL_123212), A/Moscow/10/1999 (EPI_ISL_127595), A/Victoria/361/2011 (EPI_ISL_158723), and A/Hong Kong/2671/2019 (EPI_ISL_882915). For virus rescue using the eight-plasmid reverse genetics system, transfection was performed in HEK 293T/MDCK-SIAT1 cells (Sigma-Aldrich, Cat#: 05071502-1VL) that were co-cultured (ratio of 6:1) at 60% confluence using Lipofectamine 2000 (Life Technologies) according to the manufacturer’s instructions. At 24 hr post-transfection, cells were washed twice with phosphate-buffered saline (PBS) and cell culture medium was replaced with OPTI-MEM medium supplemented with 0.8 μg mL^–1^ TPCK-trypsin. Virus was harvested at 72 hr post-transfection. For measuring virus titer by TCID_50_ assay, MDCK-SIAT1 cells were washed twice with PBS prior to the addition of virus, and OPTI-MEM medium was supplemented with 0.8 μg mL^–1^ TPCK-trypsin.

### Mutant library construction

For each mutant library, insert and vector fragments were generated by PCR using PrimeSTAR Max DNA Polymerase (Takara) according to the manufacturer’s instructions, with WT NA-encoding plasmid (pHW2000-NA) as templates. Primers for the insert contained the combinatorial mutations of interest and are shown in Supplementary file 1. For insert fragment, two rounds of PCR were performed. Forward primers (P2 set) and reverse primers (P3 set) were mixed at the indicated molar ratio and used for the first round PCR (Supplementary file 1). Products from the first round PCR were then purified using Monarch DNA Gel Extraction Kit (New England Biolabs) and used as the templates for the second round insert PCR. Forward primers (P1 set) and reverse primers (P3 set) were mixed at the indicated molar ratio and used for the second round PCR (Supplementary file 1). Of note, the same reverse primers (P3 set) were used for both rounds PCR. Primers for the vector PCR are also shown in Supplementary file 1. The final PCR products of the inserts and vectors were purified by PureLink PCR purification kit (Thermo Fisher Scientific), digested by DpnI and BsmBI (New England Biolabs), and ligated using T4 DNA ligase (New England Biolabs). The ligated product was transformed into MegaX DH10B T1R cells (Thermo Fisher Scientific). At least 1 million colonies were collected for each mutant library. Plasmid mutant libraries were purified from the bacteria colonies using Plasmid Midi Kit (QIAGEN).

### High-throughput fitness measurement of NA mutants

Each plasmid mutant library was rescued as described above (see section ‘Recombinant influenza virus’) in a T75 flask, titered by TCID_50_ assay using MDCK-SIAT1 cells then stored at –80°C until use. To passage the virus mutant libraries, MDCK-SIAT1 cells in s T75 flask were washed twice with PBS and then infected with a multiplicity of infection (MOI) of 0.02 in OPTI-MEM medium containing 0.8 µg mL^–1^ TPCK-trypsin. Infected cells were washed twice with PBS at 2 hr post-infection, then fresh OPTI-MEM medium containing 0.8 µg mL^–1^ TPCK-trypsin was added to the cells. At 24 hr post-infection, supernatant containing the virus was collected. Viral RNA was extracted using QIAamp Viral RNA Mini Kit (QIAGEN). Purified viral RNA was reverse transcribed to cDNA using Superscript III reverse transcriptase (Thermo Fisher Scientific). The adapter sequence for Illumina sequencing was added to the plasmid or cDNA from the post-passaging virus mutant libraries by PCR using sequencing library preparation primers listed in Supplementary file 1. An additional PCR was performed to add the rest of the adapter sequence and index to the amplicon using primers: 5′-AAT GAT ACG GCG ACC ACC GAG ATC TAC ACT CTT TCC CTA CAC GAC GCT-3′ and 5′-CAA GCA GAA GAC GGC ATA CGA GAT XXX XXX GTG ACT GGA GTT CAG ACG TGT GCT-3′. Positions annotated by an X represent the nucleotides for the index sequence. The final PCR products were purified by PureLink PCR purification kit (Thermo Fisher Scientific) and submitted for next-generation sequencing using Illumina MiSeq PE250.

### Sequencing data analysis

Sequencing data were obtained in FASTQ format and analyzed using custom Python scripts. Briefly, sequences were parsed by SeqIO module in BioPython (Cock et al., 2009). After trimming the primer sequences, both forward and reverse-complement of the reverse reads were translated into protein sequences. A paired-end read was then filtered and removed if the protein sequences of the forward and reverse-complement of the reverse reads did not match. Subsequently, amino acids at the residues of interest were extracted. The number of reads corresponding to each of the 864 variants was then counted. The unnormalized fitness of each variant i in each replicate was estimated as follows:

$$f_{i}={log}_{10}\frac{\left({{Output}_{Count}}_{i}+1\right)/\left({{input}_{Count}}_{i}+1\right)}{\left({{Output}_{Count}}_{WT}+1\right)/\left({{input}_{Count}}_{WT}+1\right)}$$

where the ${{Output}_{Count}}_{i}$ represents the number of reads corresponding to variant i in the post-passaging virus mutant library, and the ${{input}_{Count}}_{i}$ represents the number of reads corresponding to variant i in the plasmid mutant library. A pseudocount of 1 was added to the counts to avoid division by zero. Of note, the WT sequence of Vic11 contains a naturally rare variant T329. As a result, the WT sequence of Vic11 was not included in our mutant library design. However, due to incomplete DpnI digestion of the vector during mutant library construction, the WT sequence of Vic11 was present in the Vic11 mutant library and could be detected in the next-generation sequencing data.

The final fitness value for each mutant is

$$f_{i}={log}_{10}\left(\frac{\left({{Output}_{Count}}_{i,rep1}+1\right)/\left({{input}_{Count}}_{i,rep1}+1\right)}{\left({{Output}_{Count}}_{WT,rep1}+1\right)/\left({{input}_{Count}}_{WT,rep1}+1\right)}+\frac{\left({{Output}_{Count}}_{i,rep2}+1\right)/\left({{input}_{Count}}_{i,rep2}+1\right)}{\left({{Output}_{Count}}_{WT,rep2}+1\right)/\left({{input}_{Count}}_{WT,rep2}+1\right)}\right)$$

where *rep1* and *rep2* represent replicate 1 and replicate 2, respectively. The final fitness value of each variant is listed in Figure 2—source data 1.

### Total charge of residues of interest

Positively charged amino acids (K/R) were assigned with a charge of 1, negatively charged amino acids (D/E) were –1, and neutral amino acids were 0. The net charge of a given variant is defined as the algebraic sum of the charges of the seven residues of interest. For example, the net charge of KNEGKKL, which has three positively charged amino acids and one negatively charged amino acid, is 2.

### Modeling fitness and decomposition of interactions

We modeled variant fitness as a nonlinear function of the sum of the additive and the pairwise effects by MAVE-NN (Tareen et al., 2020). Statistical learning model was trained to estimate the latent phenotype ϕ and prediction fitness $\hat{y}$ .

The sum of the additive and the pairwise effects was defined as a latent phenotype $\phi_{pairwise}$ ,

$$\phi_{pairwise}\left(\vec{x};\vec{\theta }\right)=\theta_{0}+\sum_{l=0}^{L-1}\sum_{c}\theta_{l:c}x_{l:c}+\sum_{l=0}^{L-2}\sum_{l^{`}=l+1}^{L-1}\sum_{c,c^{`}}\theta_{l:c,l^{`}:c^{`}}x_{l:c}x_{l^{`}:c^{`}}$$

where *L* is the length of the sequences, *C* is the total number of amino acids, and $x_{l:c}=\left\{ \begin{array}{l} 1\;\;if\;\;character\;\;c\;\;occurs\;\;at\;\;positionl\\ 0\;\;otherwise, \end{array}\right.$ is the one-hot encoding of the sequence at position l when amino acid is c. $\vec{\theta }$ represents the weight of the additive and the pairwise effects. Fitness is then modeled as a nonlinear function of the sum of tanh,

$$\hat{y}=g\left(\phi ;\vec{\alpha }\right)=a+\sum_{k=0}^{K-1}b_{k}tanh\left(c_{k}\phi +d_{k}\right)$$

where K specifies the number of ‘hidden nodes’ contributing to the sum, and $\vec{\alpha }=\{a,b_{k},c_{k},d_{k}\}$ are the trainable parameters.

To train the above model, dataset was reformatted to a set of *N* observations, ${\left\{\left({\vec{x}}_{n},y_{n}\right)\right\}}_{n=1}^{N}$ , where each observation comprises sequence ${\vec{x}}_{n}$ and its fitness $y_{n}$ . Then, the dataset was randomly divided into a training set, a validation set, and a test set with a ratio of 0.64:0.16:0.2. Model was evaluated using repeated k-fold cross-validation, and hyperparameters were chosen by maximizing the R^2^ of model prediction and Pearson correlation coefficient of model parameters (Otwinowski and Nemenman, 2013). Notably, we find that the R^2^ of our model prediction is insensitive to regularization and largely depends on the quality of the variant fitness data (Figure 3—figure supplement 1).

### Estimating epistasis between charged states

Amino acids are classified according to charges. Positive (+) represents positively charged amino acids (K/R), negative (-) represents negatively charged amino acids (D/E), and neutral (n) represents the remaining amino acids. All variants of the NA antigen region are then converted into charge states, named positive, negative, and neutral for each residue. For example, –344 represents K344 and R344. The epistasis value of a given charge state is the average over the epistasis values of all amino acid variants in the specified charge state.

### Analysis of natural sequences

A total of 66,562 full-length NA protein sequences from human H3N2 were downloaded from the GISAID (http://gisaid.org) (Shu and McCauley, 2017; Supplementary file 2, Figure 1—source data 1). Amino acid sequences of NA residues 328, 329, 344, 367, 368, 369, and 370 in individual strains were extracted. Individual sequences were grouped by the year of isolation, and their mean fitness in different genetic backgrounds is plotted in Figure 2c. The human H3N2 NA protein sequences used in this study are listed in Figure 1—source data 1. The same analysis was performed on the NA homologous region of influenza A H1N1 and influenza B.

### Inference of natural coevolution score

The change in the natural frequency of the charge states (+/-/n) at residue i from year n-1 to year n was computed as

$$\Delta f\left(s_{i},n\right)=\sum_{aa_{i}\in s_{i}}f_{aa_{i},n}-\sum_{aa_{i}\in s_{i}}f_{aa_{i},n-1}$$

where $s_{i}$ is the charge states (+/-/n) at residue i, and $f_{{aa}_{i},n}$ is the natural frequency of the amino acid variant (aa) at residue i in year n. Charge state ‘+’ included amino acids K and R. Charge state ‘-’ included amino acids D and E. Charge state ‘n’ included the remaining amino acids.

All local maxima were then selected and defined as peak of frequency change using find_peaks module in SciPy. Subsequently, to evaluate the proximity of peaks among two charge states, a coevolution score was calculated as the sum of exponential weights of all pairwise peak distances from a given mutation pair $\left(i,j\right)$ :

$$E\left(i,j\right)=\sum_{k}^{N}Ce^{-d_{k}}$$

where $d_{k}$ is the time separation (in years) between two peaks and *C* is initial value. *C* = 1 if both peaks have the same sign, otherwise *C* = –1 (i.e., one peak is positive and the other is negative). Pairs of peaks with a time separation $d_{k}$ of longer than 5 years were discarded. See Figure 5—figure supplement 3 for a schematic overview of calculating the coevolution score.

### Construction of evolutionary trajectories based on the fitness data

To construct an ensemble of evolutionary trajectories using the fitness data, we assume that the same haplotype would not reappear along an evolutionary trajectory. In addition, only those seven-residue variants in our mutant libraries that had a net charge of –1, 0, or 1 were included. Besides, we only included those amino acid variants that were in our mutant libraries. An ensemble of evolutionary trajectories was constructed for all six strains of interest using their corresponding WT sequences as starting points. The number of steps in the constructed trajectory is equivalent to the number of mutations that naturally emerged following the isolation of the focal strain and prior to the point when the subsequent strains of interest were sampled. For example, there were three mutations that naturally merged between 1989 and 1999. As a result, three steps were included for the constructed evolutionary trajectory of Bei89. The fitness value at each step in the evolutionary trajectory was equivalent to that of the corresponding mutant, with the fitness of the WT set as 1 (see section ‘Sequencing data analysis’).

### Code availability

Custom Python scripts for analyzing the fitness landscape data have been deposited to https://github.com/Wangyiquan95/NA_EPI (Wu, 2021; copy archived at swh:1:rev:2126f527add1c02cd9490a520d647b0700a18a2c).

## Data Availability

Raw sequencing data have been submitted to the NIH Short Read Archive under accession number: BioProject PRJNA742436. Custom python scripts for analyzing the deep mutational scanning data have been deposited to https://github.com/Wangyiquan95/NA_EPI, (copy archived at https://archive.softwareheritage.org/swh:1:rev:2126f527add1c02cd9490a520d647b0700a18a2c). The following dataset was generated: WangY NcWu 2021Deep mutational scanning of human H3N2 influenza virus NA residues 328, 329, 344, 367, 368, 369, and 370NIH Short Read Archive BioProjectPRJNA742436

## Additional files

Supplementary file 1. Primer sequences.

Supplementary file 2. Number of human H3N2 neuraminidase (NA) sequences per year.

Transparent reporting form

## References

[bib1] Abed Y, Pizzorno A, Bouhy X, Boivin G (2011). Role of permissive neuraminidase mutations in influenza A/Brisbane/59/2007-like (H1N1) viruses. PLOS Pathogens.

[bib2] Air GM, Els MC, Brown LE, Laver WG, Webster RG (1985). Location of antigenic sites on the three-dimensional structure of the influenza N2 virus neuraminidase. Virology.

[bib3] Air GM (2012). Influenza neuraminidase. Influenza and Other Respiratory Viruses.

[bib4] Bloom JD, Labthavikul ST, Otey CR, Arnold FH (2006). Protein stability promotes evolvability. PNAS.

[bib5] Bloom JD, Gong LI, Baltimore D (2010). Permissive secondary mutations enable the evolution of influenza oseltamivir resistance. Science.

[bib6] Bloom Jesse D, Silberg JJ, Wilke CO, Drummond DA, Adami C, Arnold FH (2005). Thermodynamic prediction of protein neutrality. PNAS.

[bib7] Breen MS, Kemena C, Vlasov PK, Notredame C, Kondrashov FA (2012). Epistasis as the primary factor in molecular evolution. Nature.

[bib8] Callahan B, Neher RA, Bachtrog D, Andolfatto P, Shraiman BI (2011). Correlated evolution of nearby residues in Drosophilid proteins. PLOS Genetics.

[bib9] Cock PJA, Antao T, Chang JT, Chapman BA, Cox CJ, Dalke A, Friedberg I, Hamelryck T, Kauff F, Wilczynski B, de Hoon MJL (2009). Biopython: freely available Python tools for computational molecular biology and bioinformatics. Bioinformatics.

[bib10] Colman PM, Varghese JN, Laver WG (1983). Structure of the catalytic and antigenic sites in influenza virus neuraminidase. Nature.

[bib11] Couch RB, Atmar RL, Franco LM, Quarles JM, Wells J, Arden N, Niño D, Belmont JW (2013). Antibody correlates and predictors of immunity to naturally occurring influenza in humans and the importance of antibody to the neuraminidase. The Journal of Infectious Diseases.

[bib12] de Graff AMR, Hazoglou MJ, Dill KA (2016). Highly Charged Proteins: The Achilles’ Heel of Aging Proteomes. Structure.

[bib13] Doud MB, Ashenberg O, Bloom JD (2015). Site-Specific Amino Acid Preferences Are Mostly Conserved in Two Closely Related Protein Homologs. Molecular Biology and Evolution.

[bib14] Duan S, Govorkova EA, Bahl J, Zaraket H, Baranovich T, Seiler P, Prevost K, Webster RG, Webby RJ (2014). Epistatic interactions between neuraminidase mutations facilitated the emergence of the oseltamivir-resistant H1N1 influenza viruses. Nature Communications.

[bib15] Echave J, Wilke CO (2017). Biophysical Models of Protein Evolution: Understanding the Patterns of Evolutionary Sequence Divergence. Annual Review of Biophysics.

[bib16] Fowler DM, Fields S (2014). Deep mutational scanning: a new style of protein science. Nature Methods.

[bib17] Gong LI, Suchard MA, Bloom JD (2013). Stability-mediated epistasis constrains the evolution of an influenza protein. eLife.

[bib18] Gulati U, Hwang C-C, Venkatramani L, Gulati S, Stray SJ, Lee JT, Laver WG, Bochkarev A, Zlotnick A, Air GM (2002). Antibody epitopes on the neuraminidase of a recent H3N2 influenza virus (A/Memphis/31/98). Journal of Virology.

[bib19] Hockenberry AJ, Wilke CO (2019). Evolutionary couplings detect side-chain interactions. PeerJ.

[bib20] Hom N, Gentles L, Bloom JD, Lee KK (2019). Deep Mutational Scan of the Highly Conserved Influenza A Virus M1 Matrix Protein Reveals Substantial Intrinsic Mutational Tolerance. Journal of Virology.

[bib21] Kilbourne ED, Johansson BE, Grajower B (1990). Independent and disparate evolution in nature of influenza A virus hemagglutinin and neuraminidase glycoproteins. PNAS.

[bib22] Koel BF, Burke DF, van der Vliet S, Bestebroer TM, Rimmelzwaan GF, Osterhaus ADME, Smith DJ, Fouchier RAM (2019). Epistatic interactions can moderate the antigenic effect of substitutions in haemagglutinin of influenza H3N2 virus. The Journal of General Virology.

[bib23] Krammer F, Fouchier RAM, Eichelberger MC, Webby RJ, Shaw-Saliba K, Wan H, Wilson PC, Compans RW, Skountzou I, Monto AS (2018). NAction! How Can Neuraminidase-Based Immunity Contribute to Better Influenza Virus Vaccines?. MBio.

[bib24] Kryazhimskiy S, Dushoff J, Bazykin GA, Plotkin JB (2011). Prevalence of epistasis in the evolution of influenza A surface proteins. PLOS Genetics.

[bib25] Kumar S, Nussinov R (2002). Close-range electrostatic interactions in proteins. Chembiochem.

[bib26] Lässig M, Mustonen V, Walczak AM (2017). Predicting evolution. Nature Ecology & Evolution.

[bib27] Lee JM, Huddleston J, Doud MB, Hooper KA, Wu NC, Bedford T, Bloom JD (2018). Deep mutational scanning of hemagglutinin helps predict evolutionary fates of human H3N2 influenza variants. PNAS.

[bib28] Li C, Hatta M, Burke DF, Ping J, Zhang Y, Ozawa M, Taft AS, Das SC, Hanson AP, Song J, Imai M, Wilker PR, Watanabe T, Watanabe S, Ito M, Iwatsuki-Horimoto K, Russell CA, James SL, Skepner E, Maher EA, Neumann G, Klimov AI, Kelso A, McCauley J, Wang D, Shu Y, Odagiri T, Tashiro M, Xu X, Wentworth DE, Katz JM, Cox NJ, Smith DJ, Kawaoka Y (2016). Selection of antigenically advanced variants of seasonal influenza viruses. Nature Microbiology.

[bib29] Luksza M, Lässig M (2014). A predictive fitness model for influenza. Nature.

[bib30] Lyons DM, Lauring AS (2018). Mutation and Epistasis in Influenza Virus Evolution. Viruses.

[bib31] Magliery TJ, Regan L (2004). Beyond consensus: statistical free energies reveal hidden interactions in the design of a TPR motif. Journal of Molecular Biology.

[bib32] Malby RL, Tulip WR, Harley VR, McKimm-Breschkin JL, Laver WG, Webster RG, Colman PM (1994). The structure of a complex between the NC10 antibody and influenza virus neuraminidase and comparison with the overlapping binding site of the NC41 antibody. Structure.

[bib33] Memoli MJ, Shaw PA, Han A, Czajkowski L, Reed S, Athota R, Bristol T, Fargis S, Risos K, Powers JH, Davey RT, Taubenberger JK (2016). Evaluation of Antihemagglutinin and Antineuraminidase Antibodies as Correlates of Protection in an Influenza A/H1N1 Virus Healthy Human Challenge Model. MBio.

[bib34] Miton CM, Tokuriki N (2016). How mutational epistasis impairs predictability in protein evolution and design. Protein Science.

[bib35] Monto AS, Petrie JG, Cross RT, Johnson E, Liu M, Zhong W, Levine M, Katz JM, Ohmit SE (2015). Antibody to Influenza Virus Neuraminidase: An Independent Correlate of Protection. The Journal of Infectious Diseases.

[bib36] Mustonen V, Kinney J, Callan CG, Lässig M (2008). Energy-dependent fitness: a quantitative model for the evolution of yeast transcription factor binding sites. PNAS.

[bib37] Narayanan KK, Procko E (2021). Deep Mutational Scanning of Viral Glycoproteins and Their Host Receptors. Frontiers in Molecular Biosciences.

[bib38] Neher E (1994). How frequent are correlated changes in families of protein sequences?. PNAS.

[bib39] Neher RA, Bedford T, Daniels RS, Russell CA, Shraiman BI (2016). Prediction, dynamics, and visualization of antigenic phenotypes of seasonal influenza viruses. PNAS.

[bib40] Neumann G, Watanabe T, Ito H, Watanabe S, Goto H, Gao P, Hughes M, Perez DR, Donis R, Hoffmann E, Hobom G, Kawaoka Y (1999). Generation of influenza A viruses entirely from cloned cDNAs. PNAS.

[bib41] Nourmohammad A, Held T, Lässig M (2013). Universality and predictability in molecular quantitative genetics. Current Opinion in Genetics & Development.

[bib42] Otwinowski J, Nemenman I (2013). Genotype to phenotype mapping and the fitness landscape of the *E. coli* lac promoter. PLOS ONE.

[bib43] Otwinowski J, McCandlish DM, Plotkin JB (2018). Inferring the shape of global epistasis. PNAS.

[bib44] Raghunathan G, Sokalingam S, Soundrarajan N, Madan B, Munussami G, Lee S-G (2013). Modulation of protein stability and aggregation properties by surface charge engineering. Molecular BioSystems.

[bib45] Sandbulte MR, Westgeest KB, Gao J, Xu X, Klimov AI, Russell CA, Burke DF, Smith DJ, Fouchier RAM, Eichelberger MC (2011). Discordant antigenic drift of neuraminidase and hemagglutinin in H1N1 and H3N2 influenza viruses. PNAS.

[bib46] Shashikala HBM, Chakravorty A, Alexov E (2019). Modeling Electrostatic Force in Protein-Protein Recognition. Frontiers in Molecular Biosciences.

[bib47] Shu Y, McCauley J (2017). GISAID: Global initiative on sharing all influenza data - from vision to reality. Euro Surveillance.

[bib48] Soh YS, Moncla LH, Eguia R, Bedford T, Bloom JD (2019). Comprehensive mapping of adaptation of the avian influenza polymerase protein PB2 to humans. eLife.

[bib49] Starr TN, Thornton JW (2016). Epistasis in protein evolution. Protein Science.

[bib50] Tareen A, Ireland WT, Posfai A, McCandlish DM, Kinney JB (2020). MAVE-NN: Learning Genotype-Phenotype Maps from Multiplex Assays of Variant Effect. bioRxiv.

[bib51] Thyagarajan B, Bloom JD (2014). The inherent mutational tolerance and antigenic evolvability of influenza hemagglutinin. eLife.

[bib52] Tulip WR, Varghese JN, Laver WG, Webster RG, Colman PM (1992). Refined crystal structure of the influenza virus N9 neuraminidase-NC41 Fab complex. Journal of Molecular Biology.

[bib53] Varghese JN, Laver WG, Colman PM (1983). Structure of the influenza virus glycoprotein antigen neuraminidase at 2.9 A resolution. Nature.

[bib54] Varghese JN, Webster RG, Laver WG, Colman PM (1988). Structure of an escape mutant of glycoprotein N2 neuraminidase of influenza virus A/Tokyo/3/67 at 3 A. Journal of Molecular Biology.

[bib55] Venkatramani L, Bochkareva E, Lee JT, Gulati U, Graeme Laver W, Bochkarev A, Air GM (2006). An epidemiologically significant epitope of a 1998 human influenza virus neuraminidase forms a highly hydrated interface in the NA-antibody complex. Journal of Molecular Biology.

[bib56] Weiss CD, Wang W, Lu Y, Billings M, Eick-Cost A, Couzens L, Sanchez JL, Hawksworth AW, Seguin P, Myers CA, Forshee R, Eichelberger MC, Cooper MJ (2020). Neutralizing and Neuraminidase Antibodies Correlate With Protection Against Influenza During a Late Season A/H3N2 Outbreak Among Unvaccinated Military Recruits. Clinical Infectious Diseases.

[bib57] Westgeest KB, de Graaf M, Fourment M, Bestebroer TM, van Beek R, Spronken MIJ, de Jong JC, Rimmelzwaan GF, Russell CA, Osterhaus A, Smith GJD, Smith DJ, Fouchier RAM (2012). Genetic evolution of the neuraminidase of influenza A (H3N2) viruses from 1968 to 2009 and its correspondence to haemagglutinin evolution. The Journal of General Virology.

[bib58] Westgeest KB, Bestebroer TM, Spronken MIJ, Gao J, Couzens L, Osterhaus A, Eichelberger M, Fouchier RAM, de Graaf M (2015). Optimization of an enzyme-linked lectin assay suitable for rapid antigenic characterization of the neuraminidase of human influenza A(H3N2) viruses. Journal of Virological Methods.

[bib59] Wu NC, Young AP, Dandekar S, Wijersuriya H, Al-Mawsawi LQ, Wu TT, Sun R (2013). Systematic identification of H274Y compensatory mutations in influenza A virus neuraminidase by high-throughput screening. Journal of Virology.

[bib60] Wu NC, Du Y, Le S, Young AP, Zhang TH, Wang Y, Zhou J, Yoshizawa JM, Dong L, Li X, Wu TT, Sun R (2016). Coupling high-throughput genetics with phylogenetic information reveals an epistatic interaction on the influenza A virus M segment. BMC Genomics.

[bib61] Wu NC, Xie J, Zheng T, Nycholat CM, Grande G, Paulson JC, Lerner RA, Wilson IA (2017). Diversity of Functionally Permissive Sequences in the Receptor-Binding Site of Influenza Hemagglutinin. Cell Host & Microbe.

[bib62] Wu NC, Thompson AJ, Xie J, Lin CW, Nycholat CM, Zhu X, Lerner RA, Paulson JC, Wilson IA (2018). A complex epistatic network limits the mutational reversibility in the influenza hemagglutinin receptor-binding site. Nature Communications.

[bib63] Wu NC, Otwinowski J, Thompson AJ, Nycholat CM, Nourmohammad A, Wilson IA (2020). Major antigenic site B of human influenza H3N2 viruses has an evolving local fitness landscape. Nature Communications.

[bib64] Wu N (2021). GitHub.

[bib65] Wylie CS, Shakhnovich EI (2011). A biophysical protein folding model accounts for most mutational fitness effects in viruses. PNAS.

[bib66] Zhu X, McBride R, Nycholat CM, Yu W, Paulson JC, Wilson IA (2012). Influenza virus neuraminidases with reduced enzymatic activity that avidly bind sialic Acid receptors. Journal of Virology.

[bib67] Zhu X, Turner HL, Lang S, McBride R, Bangaru S, Gilchuk IM, Yu W, Paulson JC, Crowe JE, Ward AB, Wilson IA (2019). Structural Basis of Protection against H7N9 Influenza Virus by Human Anti-N9 Neuraminidase Antibodies. Cell Host & Microbe.

